# Functional characterization of the schizophrenia associated gene *AS3MT* identifies a role in neuronal development

**DOI:** 10.1002/ajmg.b.32905

**Published:** 2022-06-19

**Authors:** Sam J. Washer, Robert Flynn, Asami Oguro‐Ando, Eilis Hannon, Joe Burrage, Aaron Jeffries, Jonathan Mill, Emma L. Dempster

**Affiliations:** ^1^ University of Exeter College of Medicine and Health, University of Exeter Exeter UK; ^2^ Cellular Operations, Wellcome Sanger Institute, Wellcome Genome Campus Hinxton UK

**Keywords:** AS3MT, CRISPR, genetics, neuropsychiatry, RNAseq, schizophrenia

## Abstract

Genome‐wide association studies (GWAS) have identified multiple genomic regions associated with schizophrenia, although many variants reside in noncoding regions characterized by high linkage disequilibrium (LD) making the elucidation of molecular mechanisms challenging. A genomic region on chromosome 10q24 has been consistently associated with schizophrenia with risk attributed to the *AS3MT* gene. Although *AS3MT* is hypothesized to play a role in neuronal development and differentiation, work to fully understand the function of this gene has been limited. In this study we explored the function of *AS3MT* using a neuronal cell line (SH‐SY5Y). We confirm previous findings of isoform specific expression of *AS3MT* during SH‐SY5Y differentiation toward neuronal fates. Using CRISPR‐Cas9 gene editing we generated *AS3MT* knockout SH‐SY5Y cell lines and used RNA‐seq to identify significant changes in gene expression in pathways associated with neuronal development, inflammation, extracellular matrix formation, and RNA processing, including dysregulation of other genes strongly implicated in schizophrenia. We did not observe any morphological changes in cell size and neurite length following neuronal differentiation and MAP2 immunocytochemistry. These results provide novel insights into the potential role of *AS3MT* in brain development and identify pathways through which genetic variation in this region may confer risk for schizophrenia.

## 1 INTRODUCTION

Schizophrenia is a severe neuropsychiatric disorder affecting 21 million people worldwide and contributing significantly to the global burden of disease (World Health Organization, 2014; Whiteford et al., 2013). Although the precise etiology of schizophrenia is still unknown, it is hypothesized to have neurodevelopmental origins (Birnbaum & Weinberger, 2017). Twin and family studies implicate a strong role for genetic factors in the etiology of schizophrenia, with an estimated heritability of 85% (Craddock et al., 2005; Cardno & Gottesman, 2000; Sullivan et al., 2003). To date genome‐wide association studies (GWAS) have identified ~270 distinct genomic regions associated with schizophrenia (Schizophrenia Working Group of the Psychiatric Genomics Consortium, 2014; Pardiñas et al., 2018), with evidence for a substantial polygenic component within signals that fall below genome‐wide significance. Because the majority of GWAS SNPs reside in regions of strong linkage disequilibrium (LD) and do not index coding variants affecting protein structure, there remains considerable uncertainty about the causal genes and pathways involved in pathogenesis and how they are functionally regulated by schizophrenia risk variants.

One of the most robust schizophrenia GWAS signals is located on chromosome 10q24 (Duarte et al., 2016; Ripke et al., 2022), spanning a 600 kb region. Although this locus encompasses 13 protein‐coding genes, follow‐up studies have nominated *AS3MT* as the lead candidate for a role in schizophrenia. The lead schizophrenia‐associated risk variant (rs11191419) is associated with altered expression of *AS3MT* in whole fetal brain and adult hippocampus and caudate (Duarte et al., 2016), and another SNP in the region (rs7085104) is associated with differential isoform expression of *AS3MT* (Li et al., 2016). Of note, *AS3MT* was found to be associated with schizophrenia in a transcriptome‐wide association study (Gusev et al., 2018). Colocalization analyses using DNA methylation quantitative trait loci (mQTL) provide strong support for rs11191419 being associated with both schizophrenia and DNA methylation across a broad genomic region in the vicinity of *AS3MT* (Hannon et al., 2016), and suggest that the association between rs11191419 and *AS3MT* expression is mediated by DNA methylation (Hannon et al., 2016). Furthermore, DNA methylation across the *AS3MT* promoter has been shown to influence expression in vitro (Yoshinaga‐Sakurai et al., 2021). Finally, AS3MT has also been highlighted in a GWAS of Alzheimer's disease (Demichele‐sweet et al., 2018), a methylome‐wide association study (MWAS) of Alzheimer's disease and psychosis (Pishva et al., 2020), and is differentially expressed in patients with depression (Li et al., 2016). These findings suggest that dysregulation in this gene may be more broadly relevant to a range of neuropsychiatric disorders. To date, however, little is known about the role of *AS3MT* in neuronal development or brain function, and the downstream transcriptional networks it influences have not been fully elucidated.

*AS3MT* encodes an arsenite methyltransferase that methylates and detoxifies arsenic using S‐adenosyl‐l‐methionine (SAM) as a methyl donor to form intermediate compounds of monomethylated and dimethylated forms of arsenate and arsenite (Thomas et al., 2007). *AS3MT* comprises of 11 exons, which together encode a full functional transcript (*AS3MT* ^*full*^) and an alternatively spliced transcript that excludes exons 2–3 (*AS3MT* ^*d2d3*^). The primary methyltransferase protein domain is located within exons 4–6 and is present in both *AS3MT* ^*full*^ and *AS3MT* ^*d2d3*^. While the role of *AS3MT* in arsenic metabolism has been extensively studied the putative role of *AS3MT* in brain development and the individual contributions of the alternative splice variants has been limited. The *AS3MT* ^*full*^ transcript possesses arsenic methylation ability, as shown through previous work (Thomas et al., 2007; Dheeman et al., 2014), however, the *AS3MT* ^*d2d3*^ transcript does not appear to methylate arsenic despite containing the methyltransferase domain, indicating an alternative function (Li et al., 2016). While *AS3MT* expression is ubiquitous, isoform‐specific expression has been reported with *AS3MT* ^*d2d3*^ expressed twofold higher in neuronal than somatic tissue compared with *AS3MT* ^*full*^ (Li et al., 2016). The ratio of the two transcripts is highest in the hippocampus, the region which is implicated prominently in psychiatric diseases (Harrison, 2004). Further, evidence supports the role of *AS3MT* splice variants in neuropsychiatric disorders as *AS3MT* ^*d2d3*^ shows increased expression in schizophrenia patients versus controls (Li et al., 2016). This is thought to occur through schizophrenia‐associated SNPs acting as eQTLs altering the expression of the *AS3MT* ^*d2d3*^ isoform but not *AS3MT* ^full^ (Li et al., 2016). For example, rs7085104 has been identified to increase the expression of the *AS3MT* ^*d2d3*^ isoform with no effect on *AS3MT* ^*full*^ in multiple brain tissues along with alteration in dopaminergic handling (Li et al., 2016; Ambrosio et al., 2019). During neurodevelopment, *AS3MT* ^*full*^ is highly expressed before birth before decreasing postnatally, however, the *AS3MT* ^*d2d3*^ transcript remains highly expressed postnatally, further supporting a role in neurodevelopment (Li et al., 2016). This has also been shown in iPSC models where there is upregulation of *AS3MT* throughout differentiation toward neuronal fates (Li et al., 2016).

**FIGURE 1:**
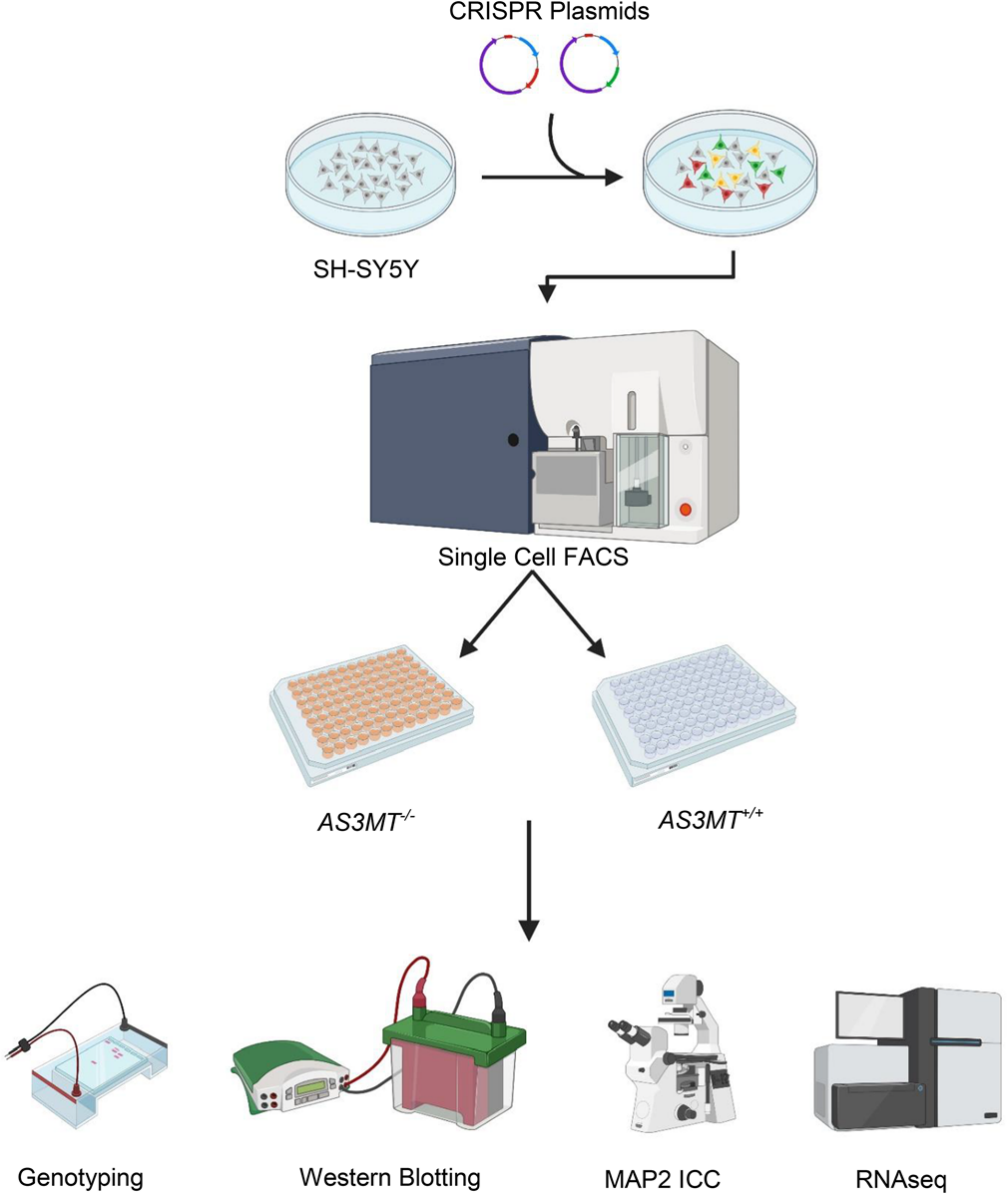
Graphical overview of the article (Image prepared in Biorender)

In this study, we used CRISPR/Cas9 to knockout AS3MT in the human SH‐SY5Y neuroblastoma cell line to investigate the role of AS3MT in neuronal cell function and explore its effect on cell morphology, *AS3MT* isoform usage, and the transcriptomic regulation of other genes in a cancer model (Figure 1). We confirm previous findings in iPSC models of isoform‐specific expression of *AS3MT* during SH‐SY5Y differentiation and show that ablation of AS3MT expression alters the activity of genes in pathways associated with neuronal development, inflammation, extracellular matrix formation, and RNA processing. Of note, we find evidence for the downstream dysregulation of other genes strongly implicated in schizophrenia.

## 2 MATERIALS AND METHODS

### 2.1 Cell culture

Human SH‐SY5Y neuroblastoma cells were maintained in T75 flasks using standard growth medium: Dulbecco's Modified Eagles Medium: Nutrient Mixture F‐12 (DMEM/F12, Gibco) supplemented with 10% (v/v) heat‐inactivated fetal bovine serum (FBS, Gibco) and cultured in a humidified atmosphere of 5% CO_2_, 37°C. Cells were passaged regularly with Accutase (Sigma) when they reached 80% confluence. For immunocytochemistry, SH‐SY5Y cells were seeded on HCl acid‐etched coverslips coated with 200 μg/ml poly‐d‐lysine (Sigma) and 20 μg/ml laminin for 24 hr (AMS Biotech). Media was changed to differentiation media: DMEM/F12 supplemented with 1% (v/v) FBS, 10 μM retinoic acid (Sigma). Differentiation media was changed every 48 hr before fixing with 4% paraformaldehyde (PFA) on Day 8.

### 2.2 AS3MT isoform quantification

SH‐SY5Y cells were seeded in six‐well plates and differentiated into a neuron‐like phenotype with retinoic acid as previously described (Teppola et al., 2016). Differentiated SH‐SY5Y cells were harvested using TRIzol (Invitrogen) followed by RNA extraction using Direct‐zol miniprep (Zymo Research), according to the manufacturer's instructions. Three biological replicates were processed for each time point. RNA was extracted at 48‐hr intervals using Trizol reagent (Invitrogen, CA, USA) and Direct‐zol RNA miniprep (Zymo Research, CA, USA) according to the manufacturer's instructions. Total RNA was converted to cDNA using the superscript VILO cDNA synthesis kit (Invitrogen) following the manufacturer's instructions. RT‐qPCR was performed on the QuantStudio 6 Flex system for *AS3MT* ^*full*^, *AS3MT* ^*d2d3*^ transcripts, and the endogenous controls β‐Actin and GAPDH with HOT FIREPol EvaGreen (Solis Biodyne, Estonia). Primer information for RT‐qPCR is listed in (Table S1). The relative expression of the two isoforms was calculated using the delta delta CT. Statistical analysis across time was performed by a one‐way ANOVA and pairwise *t*‐test post hoc analysis. Data are presented as a mean fold change relative to baseline (zero‐day) samples, error bars are ±SEM, *n* = 3 per timepoint.

### 2.3 Generation of a knockout AS3MT SH‐SY5Y cell line

**FIGURE 2:**
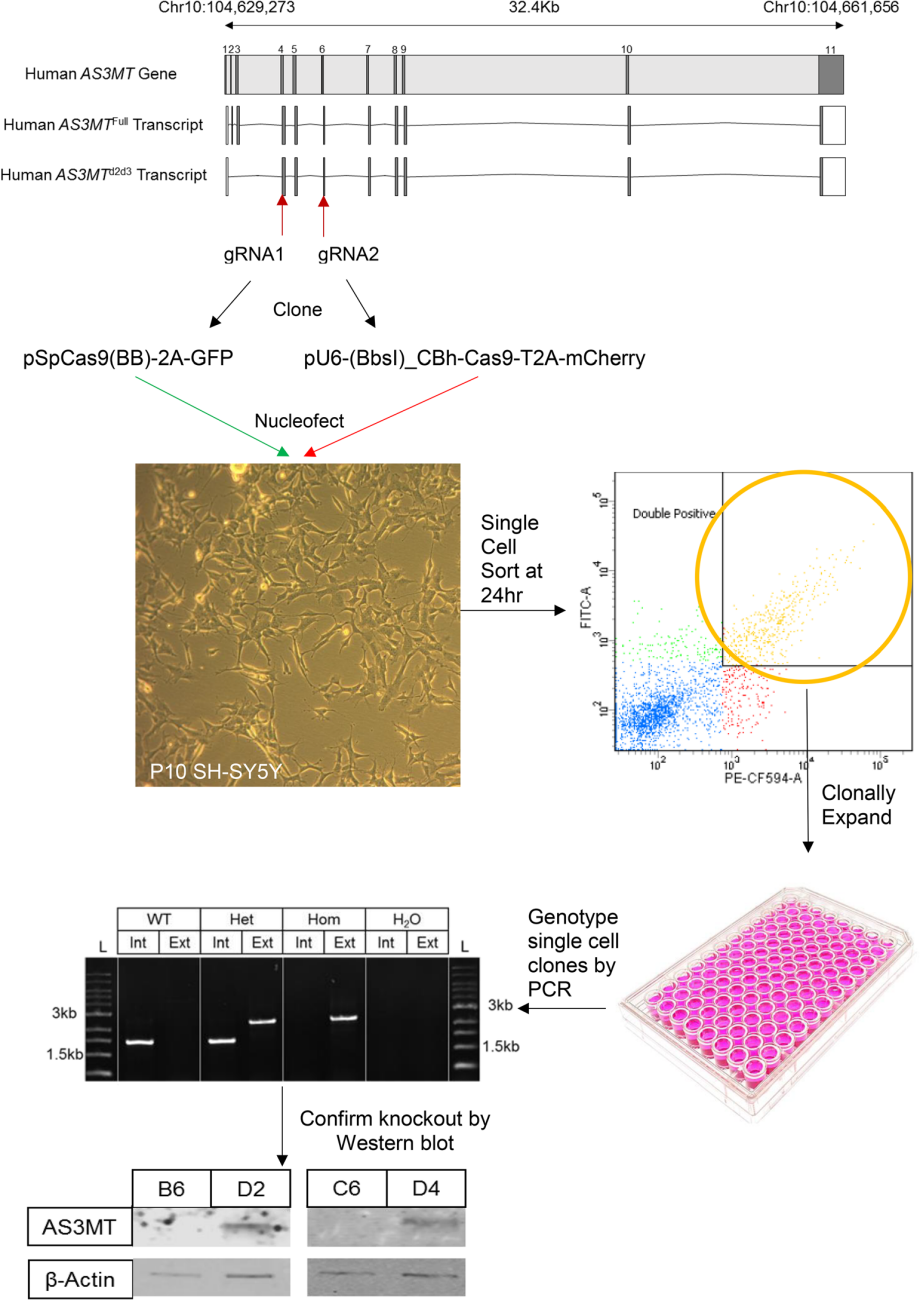
Graphical overview of the methods to generate the AS3MT knockout SH‐SY5Y cell lines used in this study: two guide RNA (gRNA) were designed to target Exon 4 and 6 of *AS3MT* to knock out both the AS3MT^d2d3^ and AS3MT^full^ transcripts. These gRNA were cloned into an all‐in‐one CRISPR vector containing a fluorescent reporter (GFP for Exon 4 and mCherry for Exon 6). These constructs were nucleofected at equimolar ratio into SH‐SY5Y cells before single‐cell sorting 24 hr post‐transfection. Single double‐positive cells were clonally expanded before genotyping for AS3MT knockout using PCR and confirmation of protein knockout by western blotting. Two AS3MT knockout lines (B6/C6) and isogenic AS3MT wild‐type lines (D2/D4) are used throughout this manuscript

AS3MT knockout SH‐SY5Y cell lines were generated to examine the transcriptional response to AS3MT disruption. When this work commenced there was no established protocol for editing SH‐SY5Y cells using CRISPR/Cas9 therefore we adopted and adapted the protocol as previously described by (Ran et al., 2013) to knockout AS3MT. We established two monoclonal homozygous knockout cell lines and age‐matched wild type control lines to proceed with transcriptional and morphological analysis. Knockout was confirmed through PCR genotyping and Western blotting (Figure 2).

The two sgRNA were designed to target the methyltransferase functional domain which is common to both isoforms using the online CRISPR design tool (http://crispr.mit.edu), and guides with the lowest off‐target score and highest on‐target scores selected. The selected guides were located in *AS3MT* exon 4 and exon 6 (Table S2). The sgRNAs were cloned into commercial CRISPR vectors pSpCas9(BB)‐2A‐GFP and pU6‐(BbsI)_CBh‐Cas9‐T2A‐mCherry which express the sgRNA, Cas9, and a fluorescent reporter (GFP or mCherry) to allow for the selection of double‐positive transfected cells. All constructs were Sanger sequenced before transfection. The final constructs used for the generation of the lines were AS3MT_Exon4_pSpCas9(BB)‐2A‐GFP and AS3MT_Exon6_pU6‐(BbsI)_CBh‐Cas9‐T2A‐mCherry. Passage 10 SH‐SY5Y cells were cultured as previously described. On the morning of electroporation, cells were harvested using accutase (Sigma) and counted using a hemocytometer. 1x10 (Sullivan et al., 2003) cells were then electroporated with 1 μg of each CRISPR construct using the SF Cell Line 4D‐Nucleofector Kit (Lonza) following the manufacturer's instructions. Nucleofection controls were included and comprised of: AS3MT_Exon4_pSpCas9(BB)‐2A‐GFP only, AS3MT_Exon6_pU6‐(BbsI)_CBh‐Cas9‐T2A‐mCherry only, pMax GFP positive control, programme negative (pMax GFP with no electroporation), substance negative (water with electroporation). The substance negative samples were used as age‐matched wild‐type (WT) controls. Following nucleofection, cells were incubated at 37°C with 5% CO_2_ for 24 hr before imaging on a conventional fluorescence microscope (Lecia), to confirm successful nucleofection (Figure S1). Double positive, mCherry and GFP cells were then sorted using fluorescent activated cell sorting (FACS). SH‐SY5Y cells were dissociated using accutase (Sigma) and single‐cell suspensions were prepared in FACS media (phenol‐free DMEM/F12 supplemented with 10% (v/v) FBS and 1x Pen/Strep). FACS was carried out using a BD Bioscience FACSAria III instrument at 20 PSI pressure using the 100 μM nozzle. GFP and mCherry double‐positive cells were single‐cell sorted into a 96‐well plate containing 500 μl of growth media with the addition of 1× pen/strep. (Figure S2). Sorted single cells were clonally expanded in 96‐well plates before passaging into 24‐well plates. At the 24‐well plate stage, cells were genotyped using PCR (Figure S3 and Table S3). Following PCR, two AS3MT knockout and two WT cell lines were maintained in culture for downstream experiments.

### 2.4 Western blotting

The abundance of AS3MT protein in the SH‐SY5Y lines was measured by Western blotting. AS3MT lines were cultured as previously described to 80% confluency before harvesting total cellular protein with lysis buffer (20 mM Tris‐Base, 150 mM NaCl, 1.25 mM EDTA, 1% Triton X) with cOmplete protease inhibitor (Roche, UK). Cell culture media was removed and cells washed with 1 ml ice‐cold PBS, 100 μl of lysis buffer containing protease inhibitor was added to each well. Cells were incubated on ice for 10 min before collection by cell scraping. Lysates were transferred to ice‐cold Eppendorfs before vortexing for 30 s. Lysates were centrifuged at 10,000 rpm for 10 min at 4°C. The supernatant was collected and quantified using a BCA assay with BSA standard controls (ThermoFisher scientific). 40 μg/ml of protein was mixed 1:1 with laemmli sample buffer and β‐mercaptoethanol (Biorad, CA, USA). Samples were heated for 5 min at 95°C. Proteins were separated using 12% SDS‐PAGE gels and transferred onto a nitrocellulose membrane (Biorad, CA, USA). Membranes were incubated in blocking buffer (TBS, 1% (v/v) Tween 20, 5% milk powder) for 1 hr on a rocker at room temperature. Membranes were then incubated in primary antibodies in blocking buffer (TBS, 1% (v/v) Tween 20, 2% milk powder) overnight at 4°C. Primary antibodies used: Mouse Anti Cyt19 (1:100, Santa‐Cruz Biotechnology) and Rabbit anti β‐actin (1:5000, NEB). Membranes were washed with TBST before the addition of secondary antibodies in blocking buffer (TBS, 1% (v/v) Tween 20, 5% milk powder) for 1 hr at room temperature. Secondary antibodies used: Goat anti‐rabbit (1:5000, Dylight 800, Invitrogen) and goat anti‐mouse (1:5000, Dylight 680, Invitrogen). Images were taken using a LI‐COR Odyssey CLx Imaging system (Figure S3).

### 2.5 Immunocytochemistry, microscopy, and image analysis

Two knockout AS3MT and two matched WT control cell lines were seeded on coverslips and differentiated into a neuronal‐like phenotype using retinoic acid for 8 days before immunocytochemistry was undertaken for MAP2 (Teppola et al., 2016; Harada et al., 2001; Korzhevskii et al., 2012), a. marker of neuronal cells in cultures of SH‐SY5Y cells (Jahn et al., 2017; Shipley et al., 2017). Differentiated AS3MT knockout and wild‐type cells were fixed with 4% paraformaldehyde (PFA) for 10 min at room temperature. Cells were permeablized with PBST (PBS, 0.16% Triton X‐100) before blocking for 1 hr with blocking buffer (PBS, 0.1% Tween‐20, 1% BSA, 300 mM glycine). Coverslips were incubated with primary antibody diluted in PBST (PBS, 0.1% Tween‐20, 1% BSA) overnight at 4°C in a humidified chamber. The primary antibody was then removed and coverslips washed three times with PBS before the addition of secondary antibody diluted in PBST for 1 hr at room temperature. Coverslips were washed three times with PBS before being mounted on glass slides using VECTASHEILD containing DAPI to counterstain the nuclei. The following antibodies were used: Mouse Anti‐MAP2 (AP20) (1:200, Santa Cruz Biotechnology, sc‐32791) and AlexaFluor 555 Goat anti Mouse (1:400, Invitrogen, A‐21422). Images were captured on a Leica DM4000 B LED Fluorescence microscope and processed with the LAS X software and ImageJ/FIJI. MAP2 positive cells were selected for analysis from three coverslips per line. The “Polygon selections” tool was used to trace the borders of MAP2‐positive cells and the area enclosed within the polygon (in μm (Whiteford et al., 2013)) was measured to determine the cell area. To determine neurite lengths, the “Segmented Line” tool was used to measure the distance (in μm) from the border of the nucleus to the distal end of neurites. The sum of the lengths of all the neurites for each cell was used to provide a total neurite length measurement for each MAP2‐positive cell. Statistical analysis was undertaken using the R statistical package (version 3.6). Statistical values stated are from a Welch's *t* test with degrees of freedom, *t*‐statistic, and *p*‐value reported. The means for genotype are reported, ±SEM.

### 2.6 RNA‐seq

RNA‐seq data was generated on two CRISPR edited AS3MT KO cell lines along with two matched control cell lines. Cells were cultured in six‐well plates until 80% confluent. Cells were harvested using TRIzol followed by RNA extraction using Direct‐zol miniprep (Zymo Research), according to the manufacturer's instructions. RNA integrity, quality, and purity were assessed using the Agilent 2200 TapeStation (Agilent). cDNA libraries were generated using the TruSeq DNA HT Library Preparation Kit (Illumina) following the manufacturer's instructions. Sequencing was performed in two batches (first experiment AS3MT KO (B6) *n* = 5, AS3MT WT(D2) *n* = 5, second experiment AS3MT KO (C6) *n* = 3, AS3MT WT (D4) *n* = 3) using the Illumina HiSeq 2,500 with 50 bp and 150 bp paired‐end reads generated for the first and second experiment, respectively.

### 2.7 RNA‐seq analysis

*Fastq* sequence quality was checked using MultiQC before alignment to the human genome (build GRCh38.p12) using STAR (Dobin et al., 2013). Mapped reads were counted at the gene level for *DESeq2* (Love et al., 2014) and at the exon level for DEXSeq (Anders et al., 2012) using the *FeatureCounts* function of the subread package (Liao et al., 2014). Differential gene expression was calculated using DESeq2, genes with a false discovery rate (FDR) *p* < 0.05 were considered differentially expressed.

To identify robust changes in gene expression between the two experiments the two data sets were meta‐analyzed using the *metagen* R package (Balduzzi et al., 2019). The meta‐analysis results and heterogeneity values were then extracted and corrected for multiple testing using a Benjamini‐Hochberg correction. Genes with a FDR < 0.01 and BH corrected heterogeneity *p* > 0.05 were taken forward for functional gene ontology analysis using GOSeq (Young et al., 2010). DEXSeq was used to examine for differential exon usage at the *AS3MT* locus to confirm knockout of the correct exons (Table S6) (Anders et al., 2012). An overview of the RNAseq quality control and analysis pipeline is shown in Figure S4).

## 3 RESULTS

### 3.1 Expression of *AS3MT* isoforms is altered through SH‐SY5Y differentiation with retinoic acid

**FIGURE 3:**
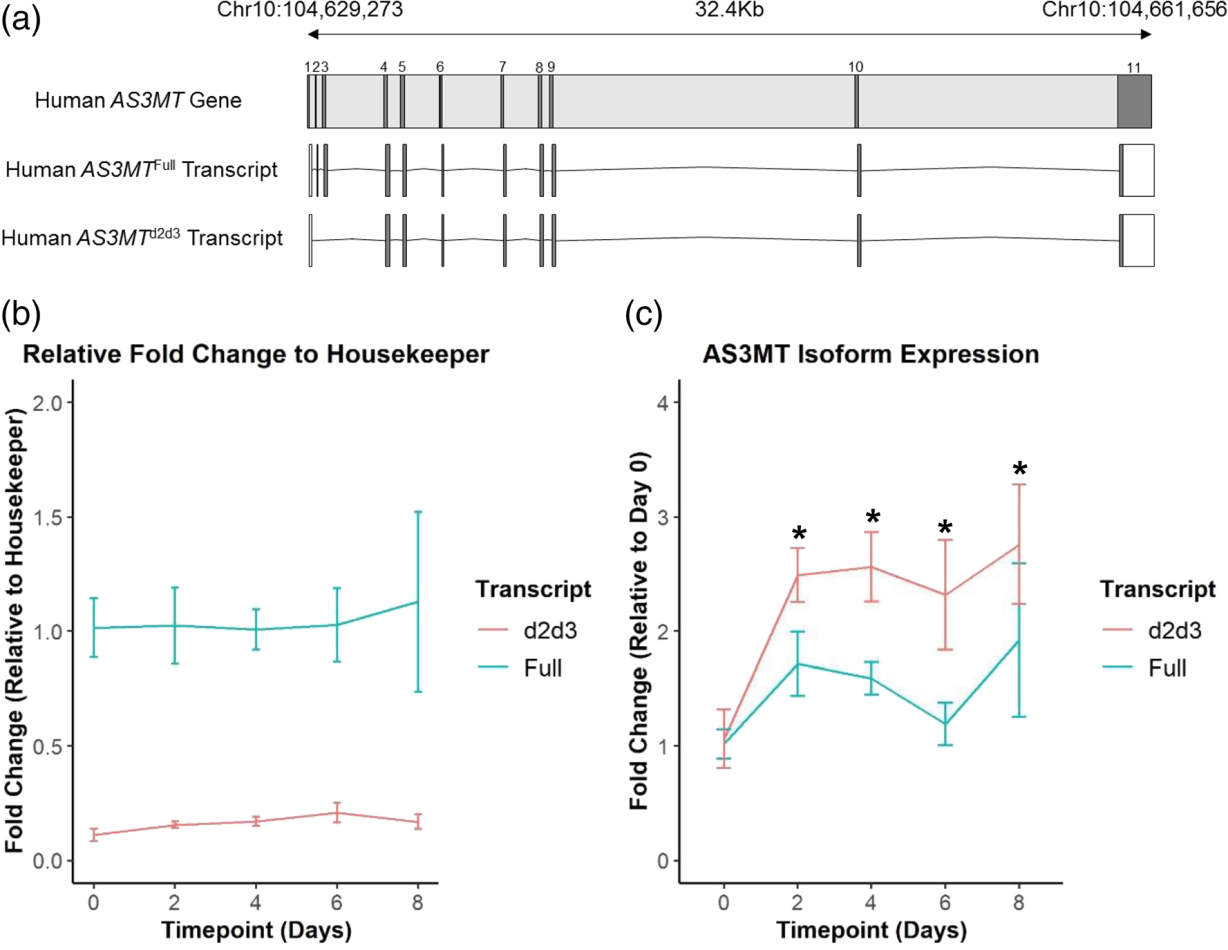
*AS3MT* isoform expression: Expression of *AS3MT* ^*d2d3*^, but not *AS3MT* ^*full*^, increases through SH‐SY5Y differentiation toward neuronal fates. SH‐SY5Y cells were differentiated to neuron‐like fates over 8 days with 10 μM retinoic acid 1% FBS media and qPCR carried out at days 0, 2, 4, 6, and 8. (a) Known isoforms of *AS3MT*. (b) Isoform expression relative to GAPDH. (c) *AS3MT* ^*full*^ expression does not change throughout differentiation (one‐way ANOVA, *F*(4, 10) =1.49, *p*‐value = 0.28). while *AS3MT* ^*d2d3*^ expression increases after neuronal differentiation initiation (one‐way ANOVA, F (4, 10) = 4.82, *p*‐value = 0.02). Normalized to day 0 timepoint

Building on previous work describing differential expression of *AS3MT* isoforms during neuronal differentiation of IPSCs (Li et al., 2016) we set out to determine if the same patterns of isoform expression occurred during the differentiation of SH‐SY5Y cells into neurons (Figure 3). We used a standard protocol to differentiate SH‐SY5Y cells toward a neuronal fate (Teppola et al., 2016). Although we found no significant change in the expression of the *AS3MT* ^*full*^ transcript (Figure 3) through neuronal‐differentiation of SH‐SY5Y cells (one‐way ANOVA, *F*(4, 10)=1.49, *p*‐value = 0.28), we identified a significant increase in the expression of the *AS3MT* ^*d2d3*^ transcript compared with baseline (day 0) (relative fold change = 2.49, one‐way ANOVA, F (4, 10)=4.82, *p*‐value = 0.02). A pairwise *t*‐test comparison identified similar significant differences in the expression of *AS3MT* ^*d2d3*^ between day 0 and day 2 (*p*‐value = 0.02), day 0 and day 4 (*p*‐value = 0.02), day 0 and day 6 (*p*‐value = 0.03), and day 0 and day 8 (*p*‐value = 0.02), suggesting an initial acute change in *AS3MT* ^*d2d3*^ that is then stably maintained (Figure 3). These results confirm the upregulation of *AS3MT* ^*d2d3*^ during the process of neuronal differentiation in SHSY5 cells, which is consistent with findings from studies of iPSCs (Li et al., 2016).

### 3.2 Confirmation of the knockout AS3MT SH‐SY5Y cell line

To identify edited cells, the expanded FACS‐isolated cell lines were PCR genotyped and Sanger sequenced, and Western blotting was performed to confirm knockout (KO) of the AS3MT protein (Figure S3). PCR analysis identified two homozygous KO cell‐lines (B6 and C6) and the 1.5Kb homozygous deletion between *AS3MT* exons 4 and 6 was confirmed by both Sanger sequencing and RNA‐seq. *AS3MT* expression was significantly decreased in both KO cell lines compared with control (B6 log_2_ fold change = −0.566, FDR *p*‐value = 2.13 x 10^−5^, C6 log_2_ fold change = −0.890, FDR *p*‐value = 2.24 x 10^−24^) (Figure S5). Examining differential exon usage with DEXSeq confirmed a significant decrease in the expression of *AS3MT* exons 5 and 6 in both KO lines compared with control (Figure S6). Stocks of the two KO lines were frozen along with two AS3MT^*+/+*^ WT lines for subsequent analyses. Our results confirm the creation of two KO AS3MT cell lines for use in functional analysis and a robust pipeline for editing SH‐SY5Y cells (Figure 2).

### 3.3 Knockout of AS3MT results in no overall changes to MAP2+ cell number, cell size, or neurite length following differentiation

**FIGURE 4:**
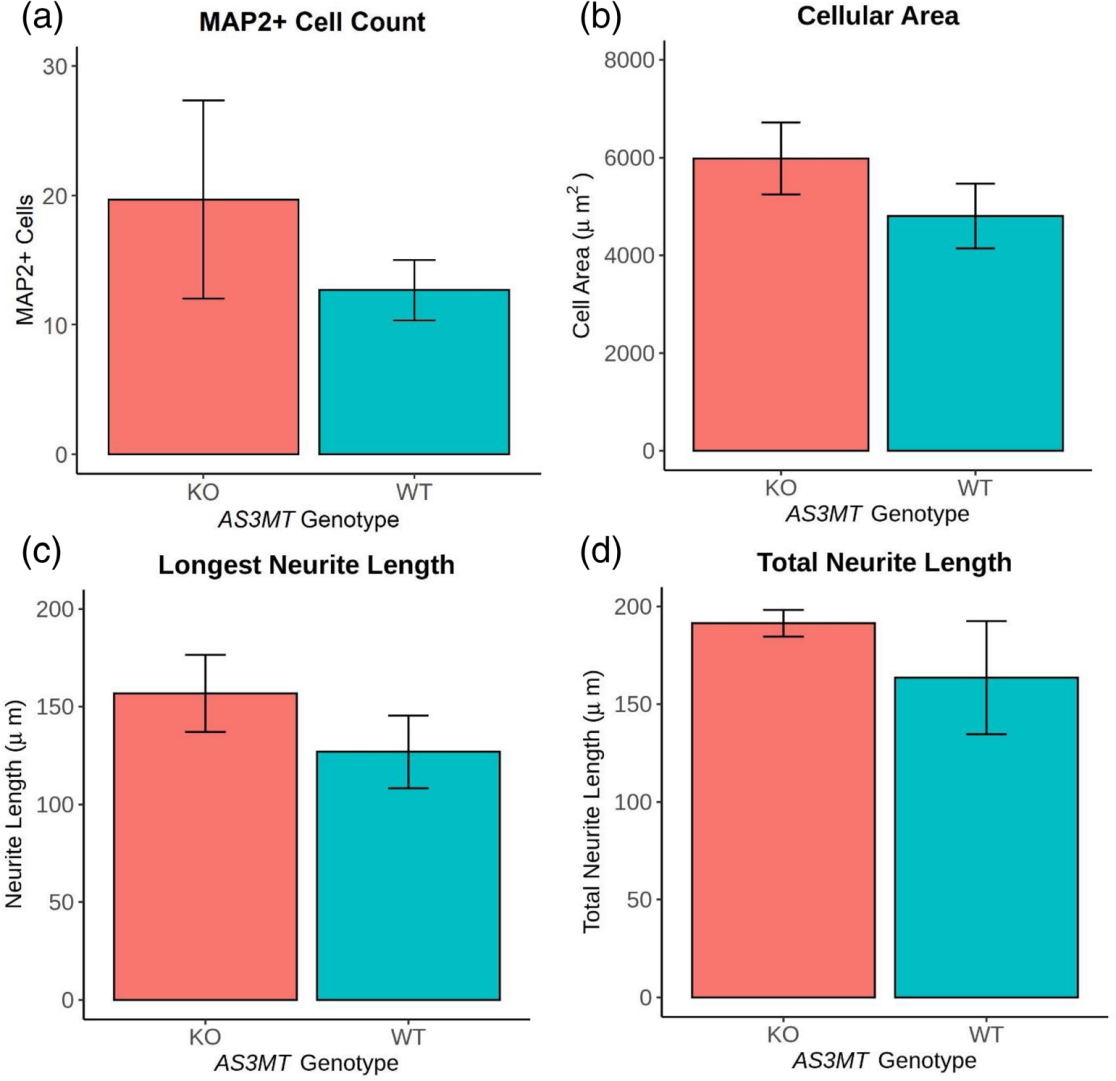
Morphology differences are not observed between differentiated AS3MT knockout and wild‐type cell lines: Cell lines were differentiated for 7 days using 10 μM retinoic acid and 1% FBS on acid‐etched, laminin and poly‐d‐lysine coated coverslips before immunocytochemistry for MAP2, a cytoskeletal marker. (a) There is no difference in the total number of MAP2+ cells between knockout (KO) or wild‐type (WT) AS3MT lines (*t* test, *t*[0.875] = 1.19, *p* = 0.523) B) There is no difference in the cellular area between KO and WT AS3MT lines (*t* test, *t*[1.98] = 1.19, *p* = 0.358). C) There is no difference in the longest neurite length between KO and WT AS3MT lines (*t* test, *t*[1.99] = 1.10, *p* = 0.386). d) There is no difference in the total neurite length between KO and WT AS3MT lines (*t* test, *t*[1.11] = 0.937, *p* = 0.508). Each experiment was carried out on two independent KO/WT cell lines with three technical repeats. Bars represent the mean of the two cell lines for each genotype. Error is mean ± SEM

After 8 days of differentiation with retinoic acid, MAP2 immunocytochemistry was performed on AS3MT KO and WT SH‐SY5Y lines to assess whether the knock‐out of AS3MT impacts upon the phenotype of differentiated neurons. MAP2 + cells were selected from three different coverslips per cell line for analysis. MAP2+ cell count, cell area, longest neurite length, and total neurite length for MAP2+ cells were calculated using neurite tracing in ImageJ (National Institute of Health, University of Wisconsin, USA) (see **Methods**). We identified no difference the total number of MAP2+ cells (*t* test, *t*[0.875] = 1.19, *p* = 0.523)cellular area (*t* test, *t*[1.98] = 1.19, *p* = 0.358), longest neurite length (*t* test, *t*[1.99] = 1.10, *p* = 0.386), or total neurite length (*t* test, *t*[1.11] = 0.937, *p* = 0.508) between the MAP2+ AS3MT KO or wild‐type cells (Figure 4). Although these results suggest AS3MT KO does not affect gross neuronal morphology it may have important downstream functional effects by dysregulation of transcriptional networks, or expression of other genes counteracts the loss of AS3MT.

### 3.4 RNA‐seq identifies consistent differential gene expression between two independent AS3MT knockout cell lines

**FIGURE 5:**
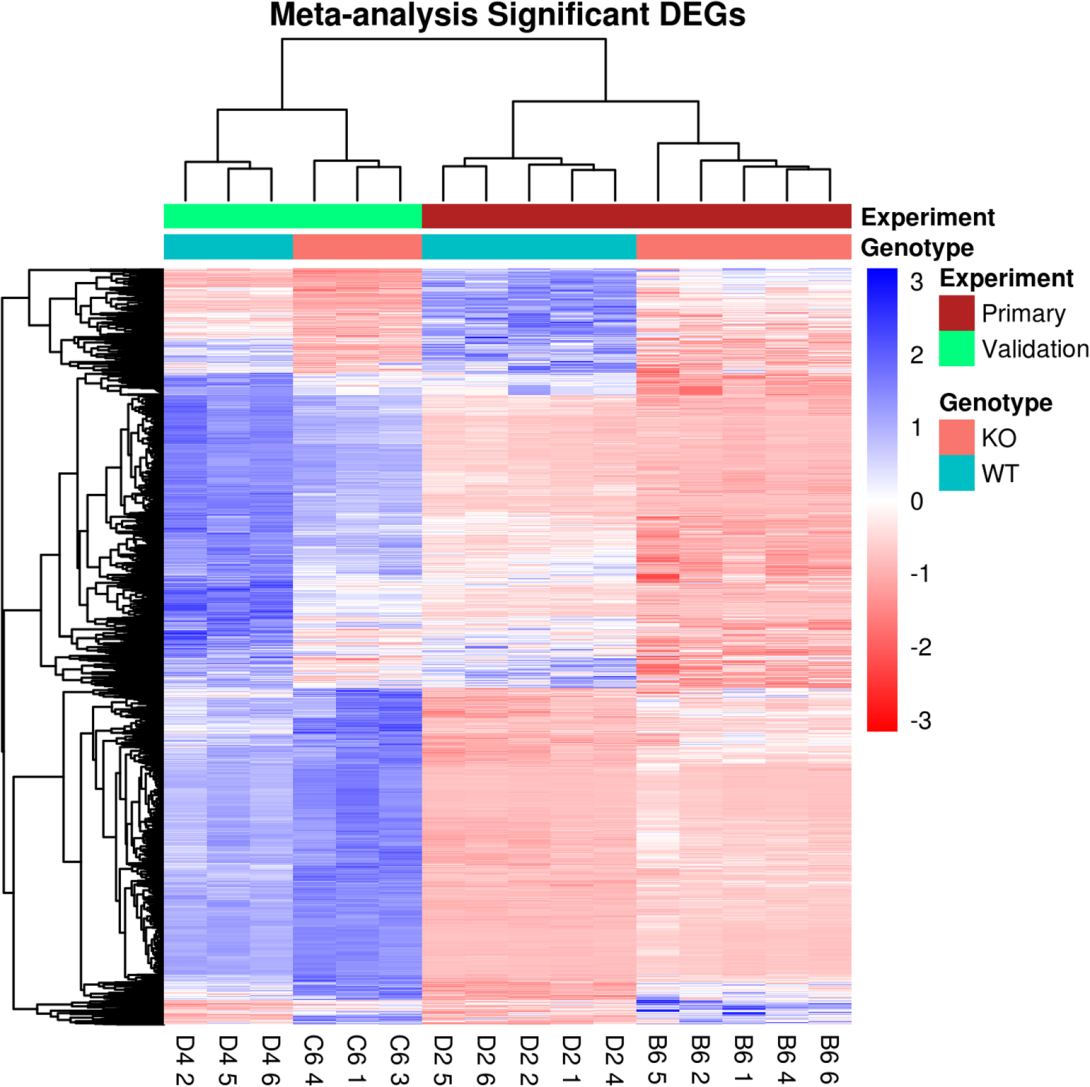
Heatmap showing the normalized counts of significant DEGs from the Meta‐analysis: The four lines underwent bulk RNAseq in two batches, including independent culturing, RNA extractions, library preparation, and sequencing run. Five technical repeats of AS3MT^−/−^ B6 and AS3MT^+/+^ D2 were run in the Primary experiment and three technical repeats of AS3MT^−/−^ C6 and AS3MT^+/+^ D4 were run in the Validation experiment. The differentially expressed genes (DEGs) from each experiment were meta‐analyzed and displayed are the normalized expression counts from the meta‐analysis of the most significant DEGs. Significance was defined by corrected *p* < 0.05 and heterogeneity *p* > 0.05. Each row is a gene scaled by the normalized gene expression where red indicates decreased count and red increased count compared with the row mean. Each column is a cell sample. The main driver of clustering is sequencing experiment, followed by genotype

In order to profile the transcriptional response to AS3MT KO we carried out paired‐end RNA‐seq on two independent AS3MT KO and WT cell lines (see Methods). Briefly, RNA‐seq reads were aligned to GRCh38.p12 using STAR, counted using feature Counts, and differential expression calculated using DESeq2. Differentially expressed genes were identified at a FDR <0.05. An overview of the RNA‐seq experiment is provided in (Figure S4). In total we identified 7,097 differentially expressed genes (DEGS) in the B6 line and 1,556 DEGS in the C6 line compared with matched WT controls (defined as FDR < 0.05) (Table S4). We found a strong correlation (*r* = 0.52) in the effect sizes for differentially expressed genes identified in the two independent KO replicates, with independent library preparation, sequencing, and control lines (764/973, exact binomial test, *p*‐value<2.2x10^−16^) (Figure S7). We subsequently meta‐analyzed the results across both experiments and identified 1,452 DEGS with a corrected *p*‐value of <0.01 and heterogeneity *p*‐value >0.05 (Table S5 and Figure 5). Of these, 804 were downregulated in the AS3MT KO cells (mean log_2_ fold change = −0.625) and 648 were upregulated (mean log_2_ fold change = +0.457). Ranking the DEGS by absolute log_2_ fold change and corrected *p*‐value the highest ranked gene was *BCHE*, which encodes for serine hydrolase enzyme butyrylcholinesterase (BChE) which was upregulated in AS3MT KO lines (log_2_ fold change +10.30, corrected *p*‐value = 1.43x10^−81^). Of note, BChE coregulates the expression of the neurotransmitter acetylcholine in the brain by hydrolyzing acetylcholine from within the synaptic cleft (Darvesh et al., 2003) and is expressed in the thalamus, amygdala, and hippocampus and is thought to play a role in nervous system development (Layer, 1991). BChE has also been implicated in schizophrenia, where increased expression of BChE was observed in ketamine dosed rats, a known model of schizophrenia, and following chronic exposure to cigarette smoke during pregnancy (Zugno et al., 2013). Furthermore, BChE has found to be upregulated in schizophrenia patients, and when treated with rivastigmine, an inhibitor of BChE and acetylcholinesterase, their quality of life was improved (Lenzi et al., 2003). The top‐ranked downregulated gene in AS3MT KO cell‐lines was *IGLON5* (log_2_ fold change −3.60, corrected *p*‐value = 3.74x10^−101^), which is part of the IgLON superfamily of neural cell adhesion molecules including *LSAMP*, *NTM*, *NEGR1*, and *OPCML* (Vanaveski et al., 2017). IgLON proteins have a diverse role, but they have been shown to regulate neurite outgrowth and synapse formation in adult and developing brain (Akeel et al., 2011; Hashimoto et al., 2009). The results from differential exon expression using DEXSeq are provided in Table S6).

### 3.5 Gene ontology analysis highlights the disruption of neurodevelopmental pathways in AS3MT KO lines

**FIGURE 6:**
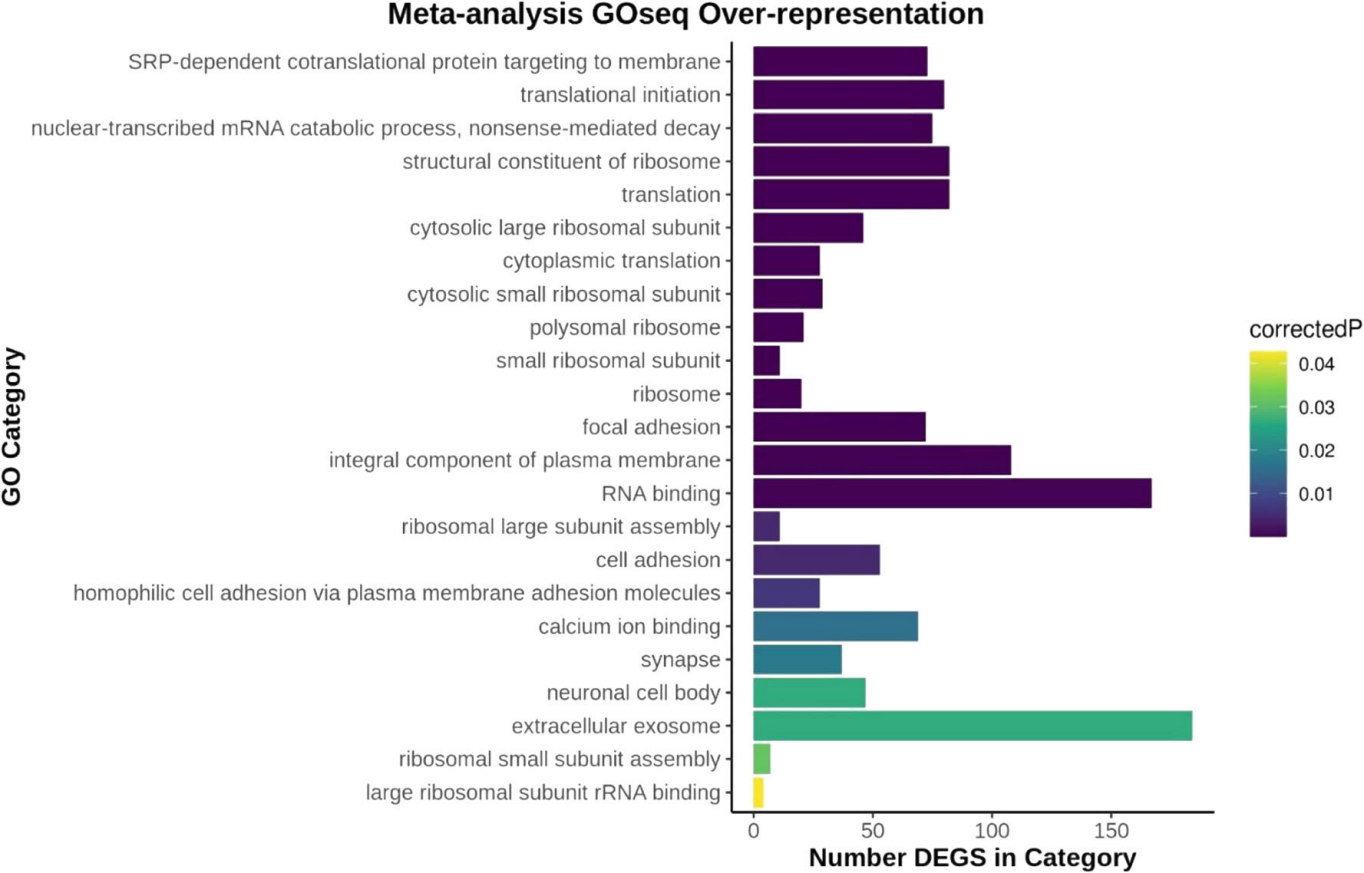
Gene Ontology analysis: GO analysis of the meta‐analysis DEGs reveals over‐representation in pathways involving translation, nonsense‐mediated decay, cell adhesion, synapse, and extracellular exosome

Biological pathways enriched amongst AS3MT KO DEGS were assessed using GOseq (see Methods). Twenty‐three GO terms were significantly overrepresented (corrected *p*‐value<0.05) amongst the 1,452 DEGS (Figure 6 and Table S7). The top pathways enriched amongst the DEGs were SRP‐dependent cotranslational protein targeting to membranes (corrected *p* < 1.49x10^−58^), translation initiation (corrected *p* < 1.13x10^−47^), and nuclear‐transcribed mRNA catabolic process nonsense‐mediated decay (NMD; corrected *p* < 3.41x10^−47^). Other relevant significant GO functions included focal adhesion (corrected *p* < 1.10x10^−6^) and cell adhesion (corrected *p* < 0.00437) indicating that cell/cell contacts are impaired following knockout. This is further supported by significant enrichment of genes involved in the synapse (corrected *p* < 0.0174), calcium ion binding (corrected *p* < 0.0161), and extracellular exosome (corrected *p* < 0.0265).

### 3.6 Significant enrichment of schizophrenia GWAS genes in DEGS following AS3MT knockout

We then set out to identify if there is an enrichment of schizophrenia‐associated genes amongst our meta‐analyzed DEGs list. Genes in schizophrenia GWAS loci were extracted from the largest and most recent GWAS of schizophrenia using a “clumping” procedure on the GWAS *p‐*values, to collapse multiple correlated signals (resulting from LD) surrounding the index SNP as described previously (Hannon et al., 2016). This analysis resulted in a total of 732 genes (Pardiñas et al., 2018), of which 492 were included in the meta‐analysis. Of these 143 were differentially expressed in AS3MT KO cells (adj *p*‐value <0.05) (Fisher's exact test: *p* = 0.0009) (Tables S8 and S9). MAGMA gene set enrichment analysis was also performed using the PGC2 SZ GWAS results to provide a further analysis of enrichment as described previously (Pardiñas et al., 2018; de Leeuw et al., 2015). Briefly, gene boundaries were expanded by 35 kb upstream and 10 kb downstream to encompass additional regulatory regions. These analyses identified modest enrichment of SZ GWAS genes in our list of differentially expressed genes (*p*‐adj <0.05) (MAGMA gene set: beta = 0.082, *p* = 2.9032e‐05).

Of note, *ZNF804A* which has been identified in multiple GWAS studies of schizophrenia (Ripke et al., 2013; O'Donovan et al., 2008), was one of the top differentially expressed genes (log_2_ fold change +1.26 corrected *p* < 3.32x10^−19^). Knockdown of *ZNF804A* in SH‐SY5Y cells alters mRNA processing involved in nervous system development and has been shown to regulate dendritic spine formation and structure (Chapman et al., 2018; Deans et al., 2017; Zhou et al., 2020) and to alter cellular adhesion (Hill et al., 2012). We also identify significant downregulation of the dopamine D2 receptor gene (*DRD2)* (Quintana & Beaulieu, 2019) (log_2_ fold change −1.15, corrected *p* < 1.52x10^−12^). Interestingly, the expression of *DRD2* is repressed by increased *ZNF804A* expression (Girgenti et al., 2012), which is consistent with our findings. Our results show an enrichment of the dysregulation of genes involved in schizophrenia, neuronal development, and cellular adhesion following knockout of AS3MT.

## 4 DISCUSSION

In this study, we used *CRISPR‐Cas9* gene editing to KO and functionally characterize the schizophrenia‐associated gene *AS3MT* in a neuronal‐like cell line. AS3MT has been extensively studied for its role in the detoxification of arsenic, although little is known about its function in neurons despite AS3MT being highly expressed during brain development and genetic variation within the gene being robustly associated with schizophrenia (Schizophrenia Working Group of the Psychiatric Genomics Consortium, 2014; Pardiñas et al., 2018; Duarte et al., 2016). Although no effects on neuronal morphology were observed in AS3MT KO lines after differentiation, we show that ablation of AS3MT expression alters the activity of genes overrepresented in genetic loci associated with schizophrenia and in pathways associated with neuronal development, inflammation, extracellular matrix formation and RNA processing. We confirm previous findings of isoform‐specific expression of *AS3MT* during neuronal differentiation. Together, our results provide novel insights into the potential role of AS3MT in brain development and identify pathways through which genetic variation in this region may confer risk for schizophrenia.

Although our study was only performed on a relatively small number of cell‐lines, the strong correlation of DEGS between our two independent AS3MT knockout lines provides confidence that the DEGS identified are robustly associated with AS3MT knockout. Pathway analysis to determine the functional relevance of the 1,452 DEGS identified from the meta‐analysis showed overrepresentation of functions involved in protein targeting, translation, cell adhesion and cell–cell interactions, neuron development and immune system responses and NMD. The enrichment of genes involved in NMD is consistent with the creation of a CRISPR induced frameshift mutation and the subsequent degradation of faulty *AS3MT* mRNA (Lykke‐andersen & Jensen, 2015; Popp & Maquat, 2016). This suggests that while *AS3MT* mRNA is still being transcribed (as confirmed by our RNAseq data) the edited mRNA is degraded and therefore not translated into a truncated protein, which is also supported by ablation of AS3MT following western blotting, therefore we are confident that NMD has resulted in KO of the functional protein. Other GO pathways enriched amongst our DEGs include focal adhesion and cell adhesion indicating that cell/cell contacts are impaired following knockout. Cellular adhesions are known to play a critical role in neurodevelopment, with roles in modulating neuronal morphology, cellular signaling and communication, axon guidance, and neuronal plasticity (Gnanapavan & Giovannoni, 2013; de Agustín‐Durán et al., 2021; Benson et al., 2000).

The enrichment of previously associated schizophrenia genes from GWAS in the list of DEGS is particularly interesting as it provides an insight into the potential interplay of these genes. For example, the identification of *ZNF804a*, and *DRD2*, two key schizophrenia risk genes, as being differentially expressed following AS3MT KO illustrates the complex polygenic architecture of this disease, where absence of one SZ susceptibility gene results in changes in other SZ susceptibility genes. Our results suggest that AS3MT is placed within a network of genes involved in brain development and schizophrenia risk. Indeed, in this study we observed a significant increase in expression of the novel isoform AS3MT^d2d3^ during differentiation mirroring previous studies in iPSC derived neurons validating our choice of model (Li et al., 2016). It is worth noting that we only examined these two isoforms of AS3MT during differentiation with retinoic acid over 8 days. Future work should examine AS3MT expression over a longer time course with other differentiation protocols (such as BDNF) and profile novel isoforms by long‐read RNA sequencing (de Medeiros et al., 2019).

One limitation of our study is the use of the single genetic background SH‐SY5Y neuroblastoma line, while this line is a valid model widely used in neuropsychiatric research due to its dopaminergic nature, reproducibility, and cost compared with iPSC models (Xicoy et al., 2017). SH‐SY5Y are also transcriptionally relevant for modeling early neurodevelopment (Chiocchetti et al., 2016). Future work should aim to validate these findings by utilizing iPSC models with different genetic backgrounds, particularly of high and low polygenic risk for schizophrenia. Once AS3MT is KO in these lines its role at synaptic and cellular junctions can be confirmed by either the differentiation into dopaminergic neurons or by creating organoids to model early neuronal development (Lancaster et al., 2013; Brennand et al., 2011).

Based on our results, we hypothesis that the *AS3MT* ^*d2d3*^ isoform plays a role in neuronal development and neuronal plasticity and is a good target for further functional validation in schizophrenia. While we observed no changes in the gross morphology of cells following AS3MT KO we note strong transcriptional differences across two independent knockout cells lines in cell adhesion and synaptic transmission, indicating that AS3MT potentially works at a subcellular level to modulate synaptic transmission rather than affecting the overall morphology of the cells. We also identified expression changes in key genes previously associated with schizophrenia such as *ZNF804A* and *DRD2*, indicating that AS3MT is a key regulator of both these genes.

## 5 CONCLUSION

We have successfully created a knockout of the schizophrenia candidate gene AS3MT in the SH‐SY5Y neuronal cell line. Our results confirm that this cell‐line can be successfully edited using CRISPR/Cas9 for genetic studies and highlighted robust disruption to the downstream expression of genes involved in synaptic transmission and cell adhesion pathways and genes previously associated with schizophrenia. These observations indicate that AS3MT works at a subcellular level to modulate synaptic transmission, providing clues to how variation in this gene increases risk for schizophrenia.

## AUTHOR CONTRIBUTIONS

S.W. and R.F. performed all experiments. S.W. analyzed the data. A.O.‐A. provided experimental insight. E.H./A.J. provided statistical and computational insight. J.B. provided technical support. S.W., A.J., J.M., and E.D. conceived and planned experiments and prepared the manuscript. All authors discussed results and contributed to the final article.

## FUNDING INFORMATION

This work was funded by an Academy of Medical Sciences Springboard grant awarded to E.L.D. (SBF001\1011). E.L.D., E.H., and J.M. were supported by Medical Research Council (MRC) grants (MR/K013807/1 and MR/R005176/1) to J.M. This project utilized equipment funded by the Wellcome Trust Institutional Strategic Support Fund (WT097835MF), Wellcome Trust Multi User Equipment Award (WT101650MA) and BBSRC LOLA award (BB/K003240/1). S.J.W., R.F., A.O.‐A, J.B., and A.J. report no biomedical financial interests or potential conflicts of interest.

## CONFLICT OF INTEREST

The authors declare no competing interests.

## ETHICS STATEMENT

Ethical approval was not required as this research utilized immortalized cell lines purchased from ATCC (www.atcc.org).

## Supporting information


**Figure S1**Supporting information

**Figure S2**Supporting Information

**Figure S3**Supporting Information

**Figure S4**Supporting Information

**Figure S5**Supporting Information

**Figure S6**Supporting Information

**Figure S7**Supporting Information

**Table S1**Supporting information

**Table S2**Supporting Information

**Table S3**Supporting Information

**Table S4**Supporting Information

**Table S5**Supporting Information

**Table S6**Supporting Information

**Table S7**Supporting Information

**Table S8**Supporting Information

**Table S9**Supporting Information

## Data Availability

All raw RNAseq files are available at GSE182157 and generated cell lines available on request.
