# Supplementary material for: Functional characterization of the schizophrenia associated gene AS3MT identifies a role in neuronal development
Source: Am J Med Genet B Neuropsychiatr Genet. 2022 Jun 19;189(5):151–62. doi: 10.1002/ajmg.b.32905 (PMC9546433; doi:10.1002/ajmg.b.32905)
Supplement: Supplementary file 1 — Figure S1Supporting information [file AJMG-189-151-s002.docx]

**SUPPLEMENTARY FIGURES**

**Supplementary Figure 1:** SH-SY5Y cells 24hr post nucleofection: Images confirm successful nucleofection and expression of CRISPR constructs. Exon 4 gRNA cloned into pSpCas9(BB)-2A-GFP and Exon 6 gRNA cloned into pU6-(BbsI)_CBh-Cas9-T2A-mCherry Imaged at 20x magnification on Leica DMi8 inverted widefield microscope. Scale bar 100μM.


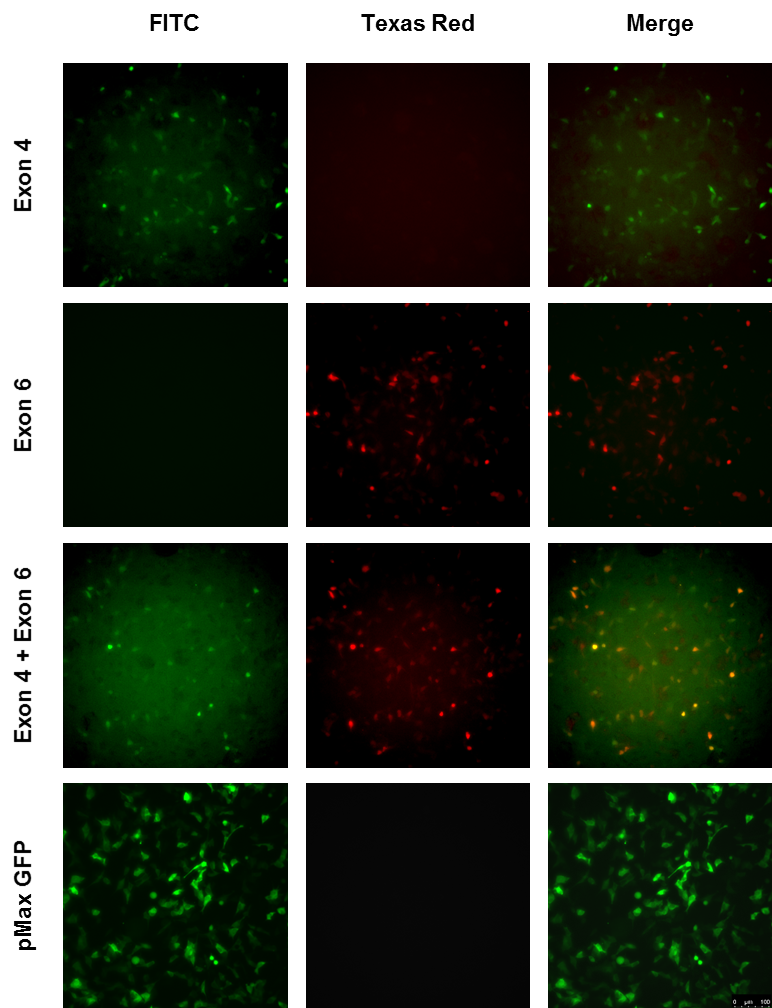


**Supplementary Figure 2:** **FACS readouts from single cell sorted transfected SH-SY5Y cell 24hr post nucleofection:** Each panel represents an individual transfection. A) Single transfected Exon 4 construct control, used for gating GFP. B) Single transfected Exon 6 construct control used for gating mCherry. C) Substance negative nucleofection control, used for gating FSC and SSC. D) Double transfected Exon 4 and Exon 6 constructs. Single cells sorted at collected from double positive population. E) Enrichment, previous sorted cells from D) were rerun through the FACS to gauge sensitivity. Single cells collected from double positive population.


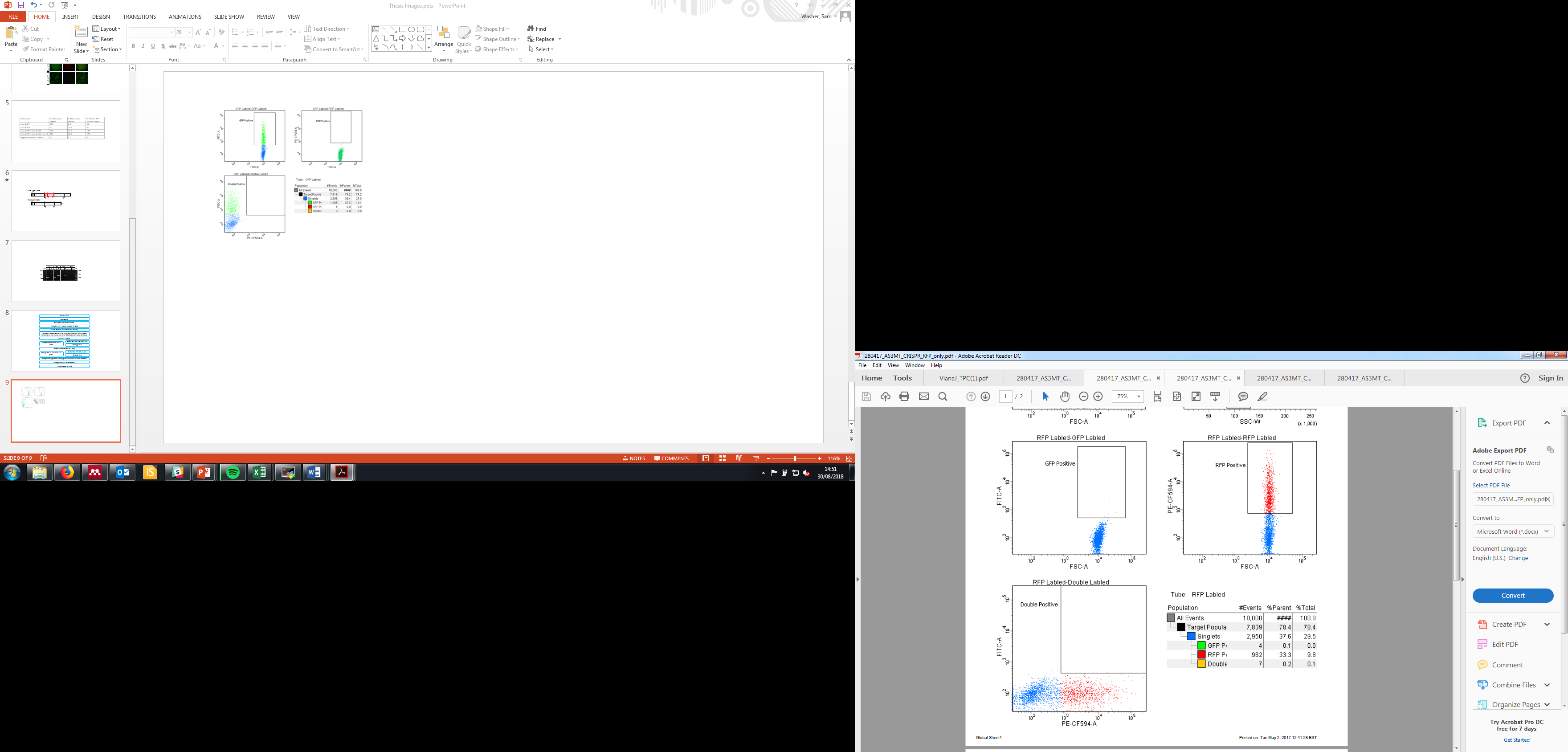

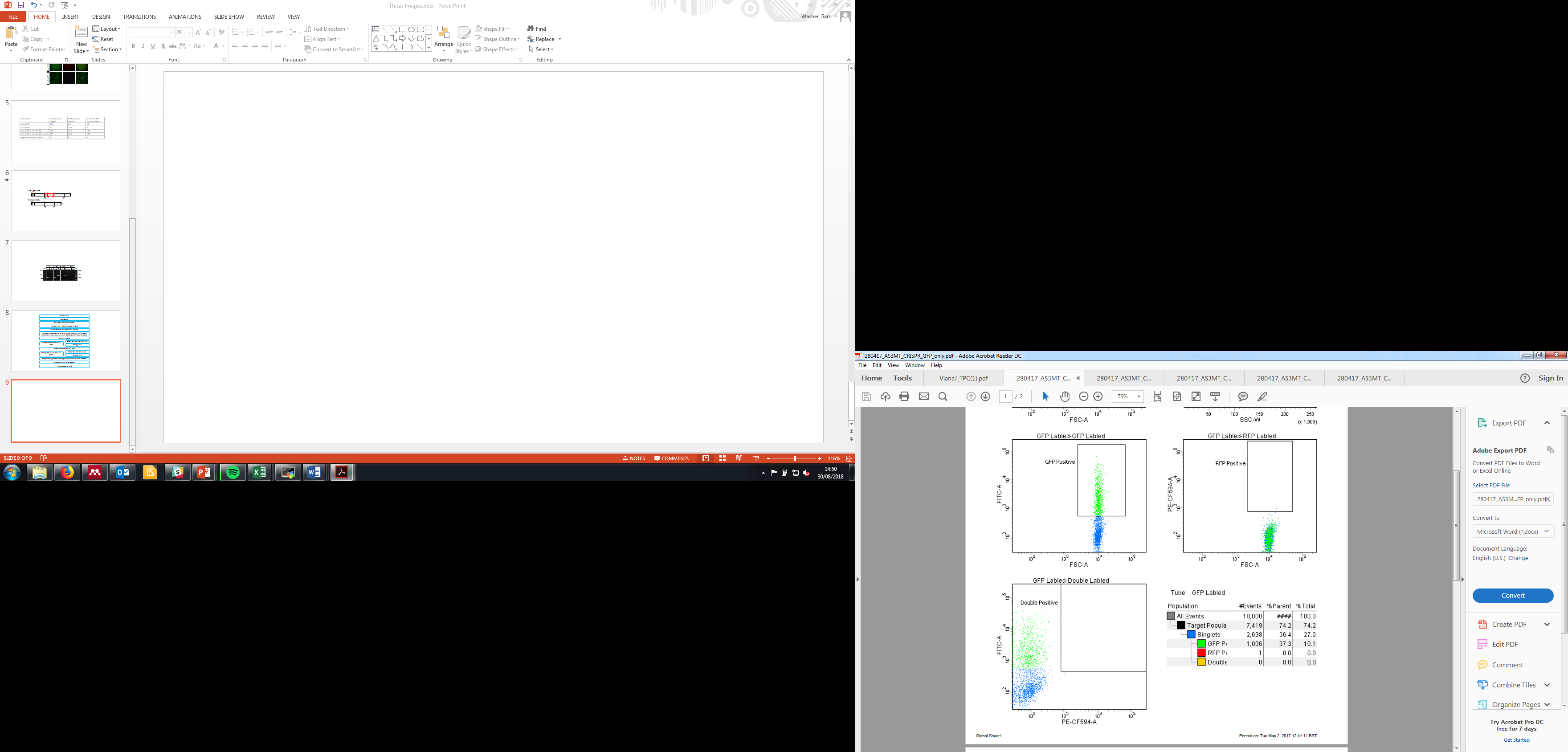

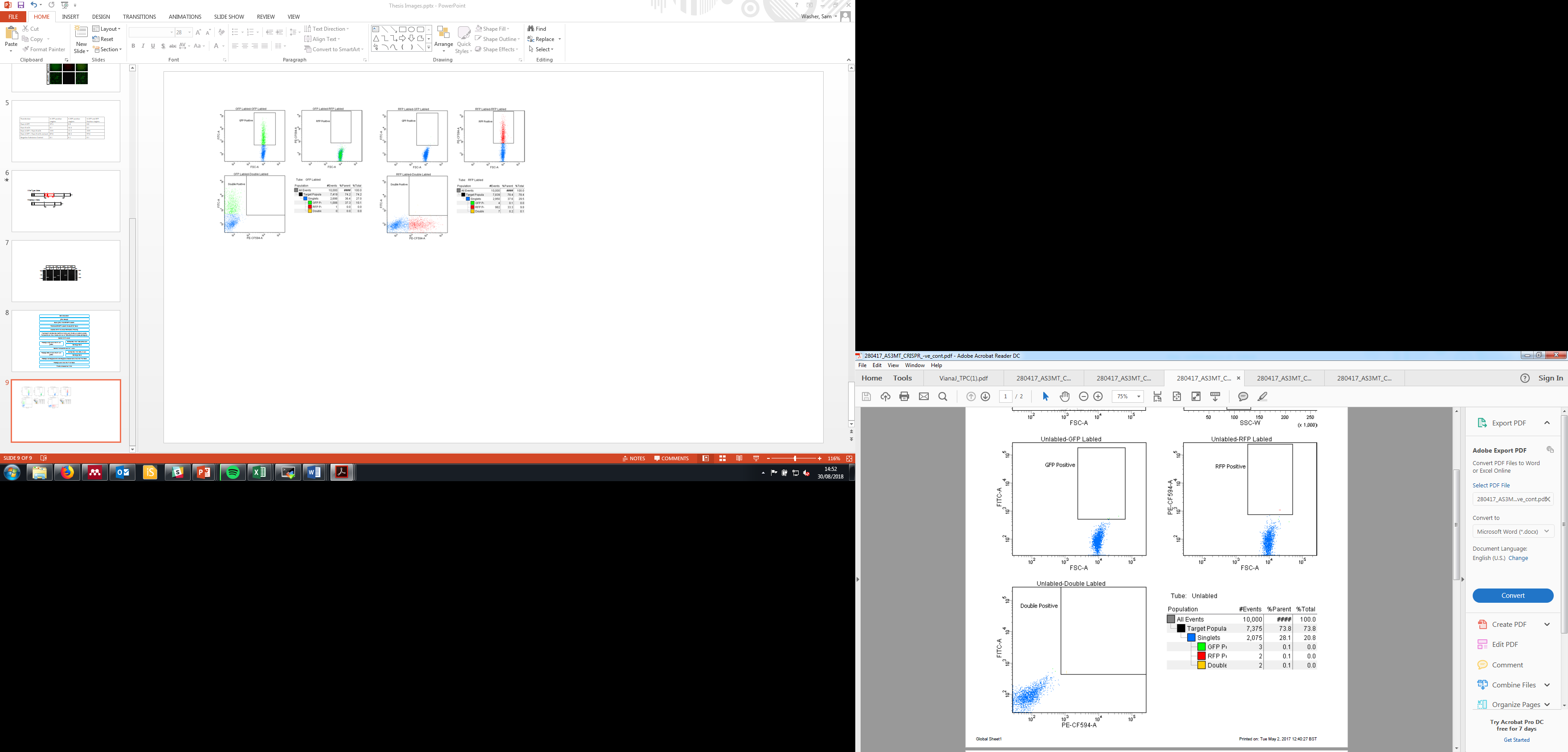

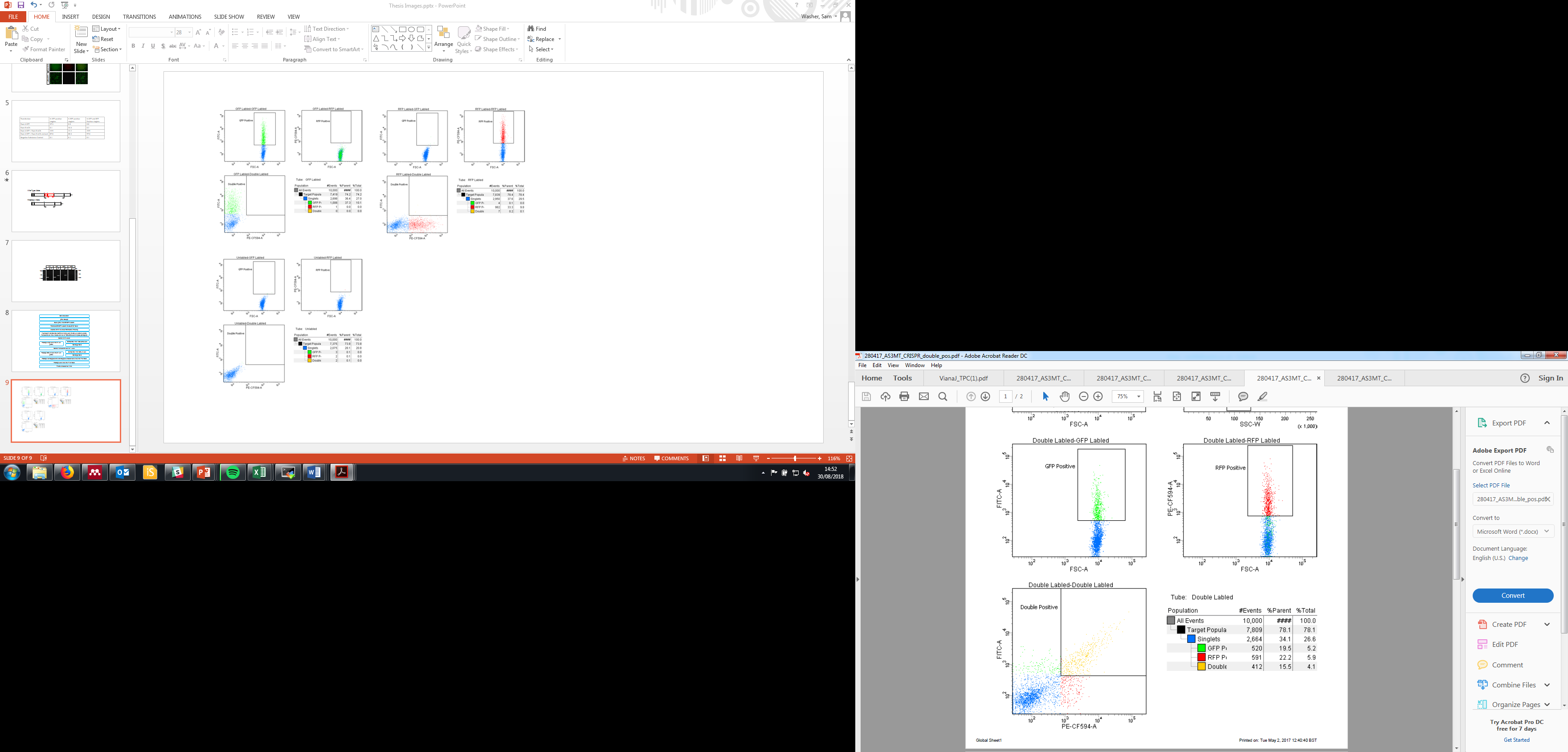


D

A

B

C

E


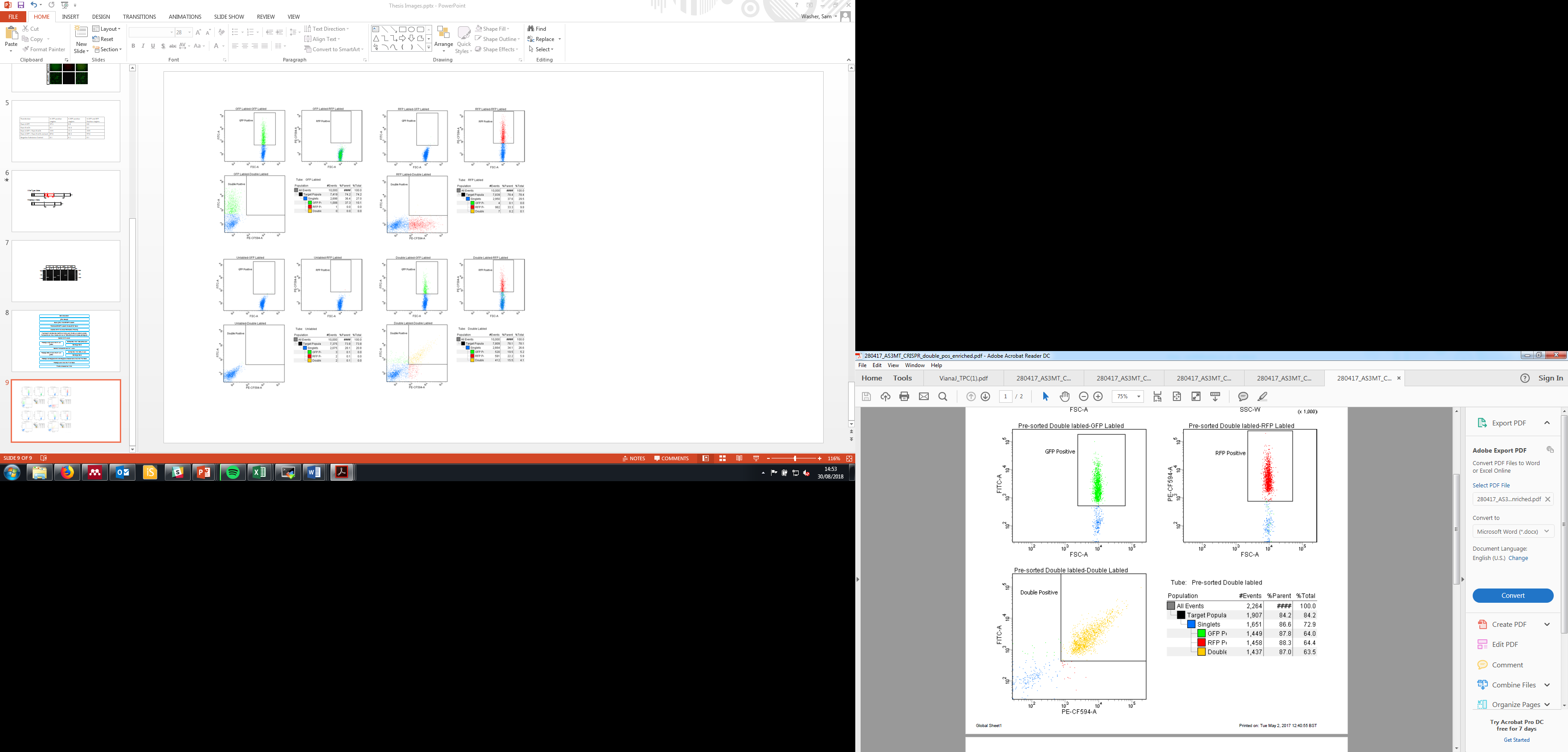


**Supplementary Figure 3: PCR Genotyping, Sanger sequencing, and western blotting confirmed knockout of AS3MT**: **A)** A representative gel image of how PCR can distinguish AS3MT genotypes. Two PCR were undertaken, one with primers within the deletion site which form a 1.5Kb product if WT or no product if KO. The other PCR reaction consisted of primers homologous to outside the deletion site creating a 4.5Kb product if WT or a 3Kb band if KO. This double PCR approach can determine WT cells (1.5Kb + 4.5Kb bands), Heterozygous (1.5Kb + 3.5Kb + 4.5Kb), and Homozygous (no band + 3.5Kb) lines. **B)** Genotyping of the FACS monoclonal lines. Double 2.6 (B6), Enriched 3 (C4), and Enriched 6 (C6) were selected as AS3MT KO lines. Non-edited controls (Sub-ve) 1 (D2), 2 (D3) and 3 (D4) were used as AS3MT WT lines. L is 1Kb ladder, the +ve control is a well containing 100 sorted double positive cells which acts as a control to check for gRNA efficiency so is a polyclonal pool. –ve control is water replacing DNA. **C)** Sanger sequencing chromatograms of the External PCR product of AS3MT KO Lines B6 and C6 confirm frame shift mutations adjacent to the PAM sequence (red) of Exon 4 gRNA (green), cut site indicated by the red arrow as determined by overlapping sequences. Sequencing for C4 was inconclusive **D)** Western blotting for AS3MT confirmed absence of AS3MT protein in all three homozygous lines. Lines C4 and D3 were not used for further experiments due to morphology differences compared to B6/D2 and C6/D4 lines.


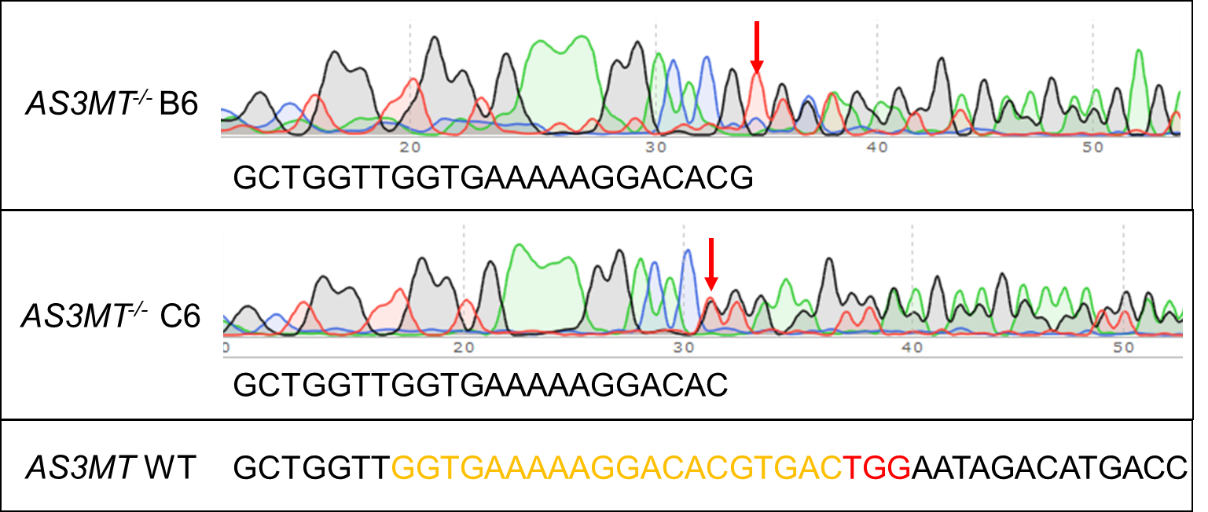

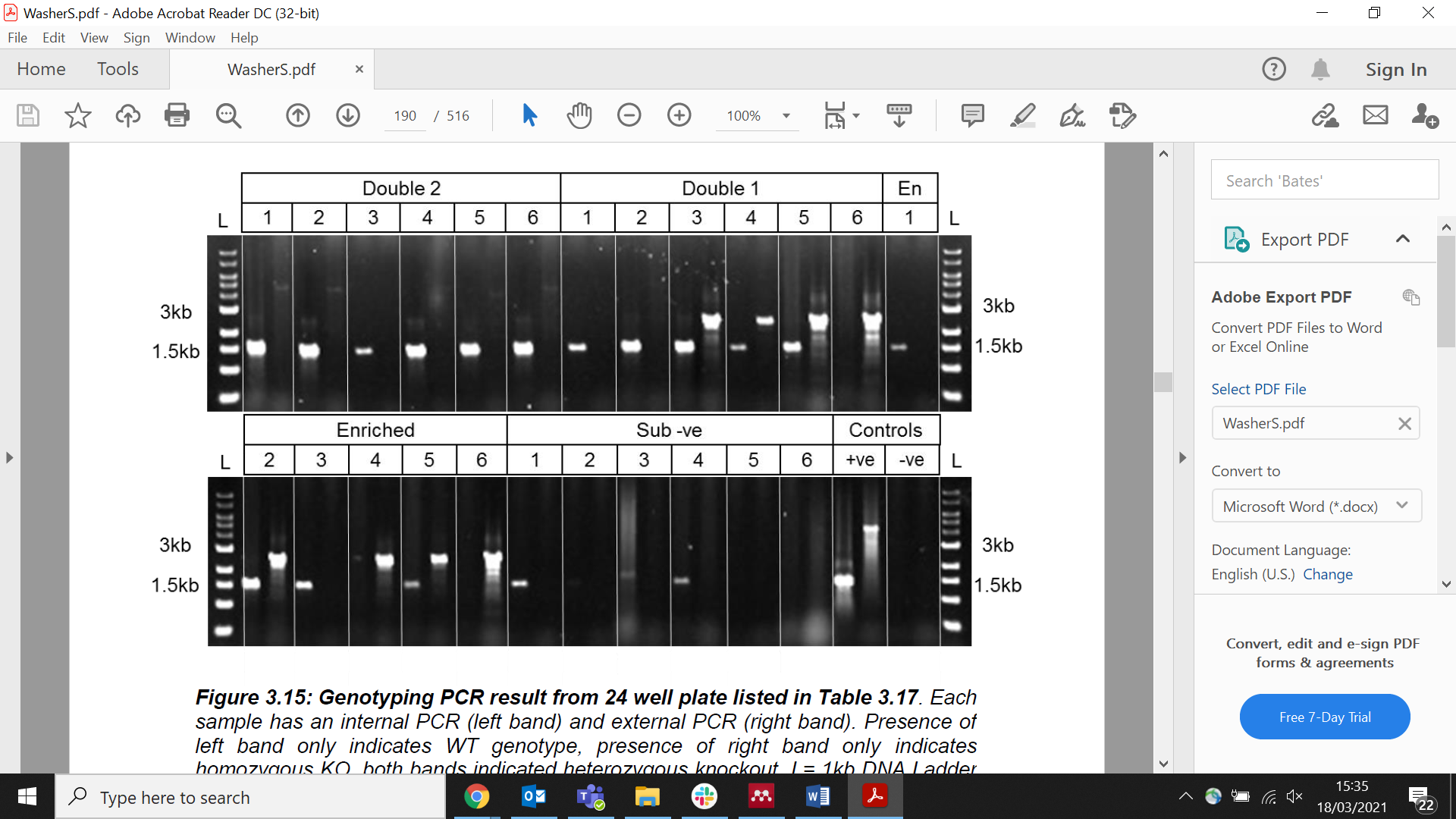

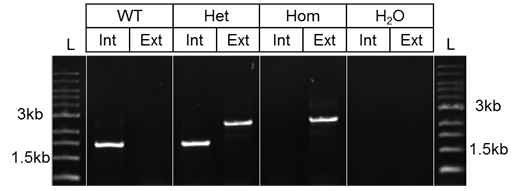


C

B

A


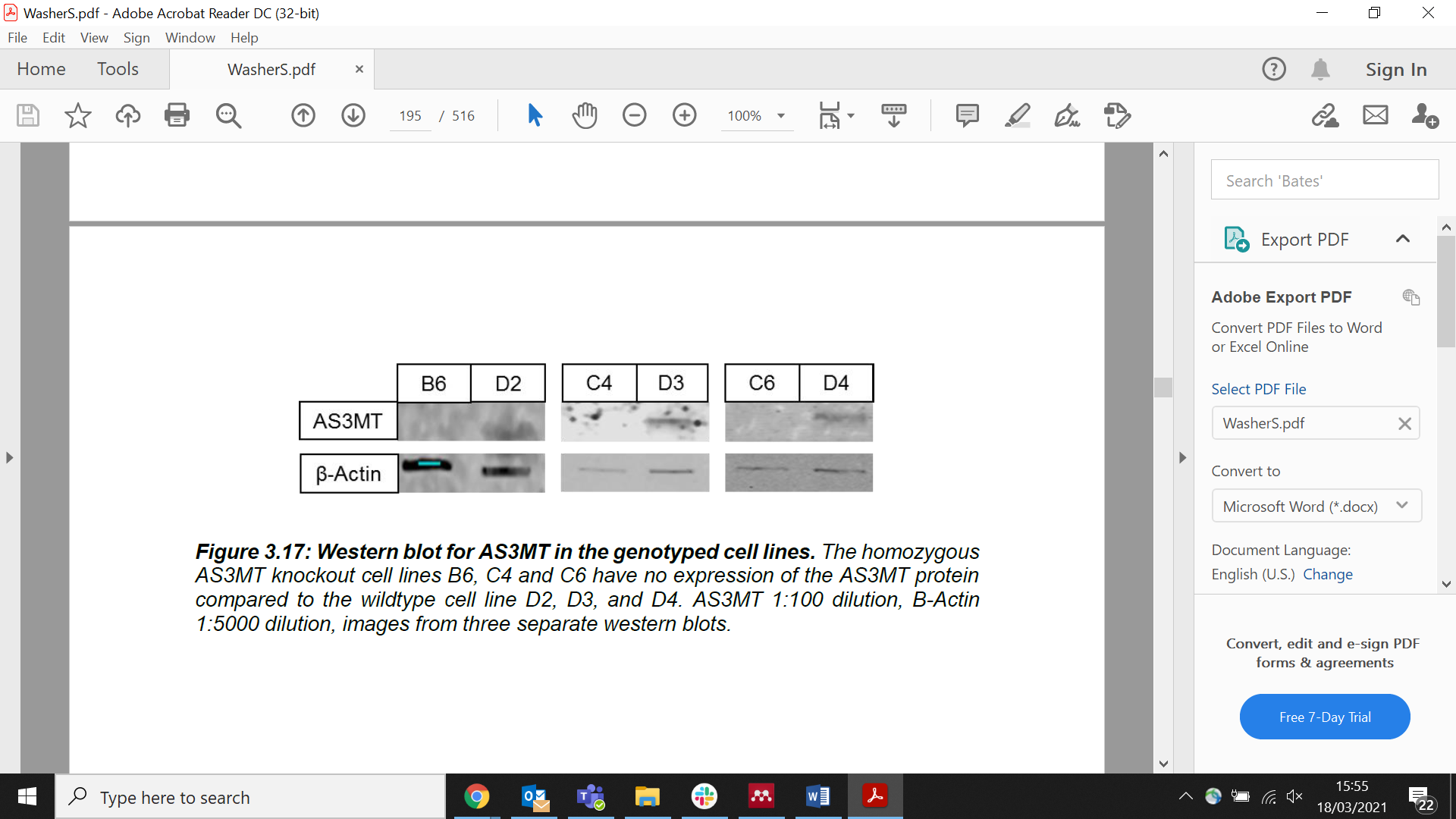


D

**Supplementary Figure 4:** A summary of the RNAseq analysis undertaken to examine transcriptional response to AS3MT knockout.

|  | **Discovery Experiment** | **Validation Experiment** |
| --- | --- | --- |
| **Cell lines** | AS3MT^-/-^ B6  AS3MT^+/+^ D2 | AS3MT^-/-^ C6  AS3MT^+/+^ D4 |
| **RNA Preparation** | Cells grown to 80% confluency in 6 well plates  RNA extraction using Directzol RNA miniprep  Quality assured by Agilent Tapestation | |
| **Library Preparation** | Samples per group: 5  RNA Integrity > 8 | Samples per group: 3  RNA Integrity > 8 |
| **Sequencing** | Read Length: 50bp  Paired End  >9 million reads / sample  Phred (Q) > 25  Mapping > 90% | Read Length: 125bp  Paired End  >20 million reads / sample  Phred (Q) > 25  Mapping > 90% |
| **Quality control & pre-analysis** | MultiQC [quality control]  Cutadapt [trimming]  STAR [mapping] | MultiQC [quality control]  Cutadapt [trimming]  STAR [mapping] |
| **Data analysis** | featureCounts [expression quantification]  DESeq2 [differential expression analysis] | featureCounts [expression quantification]  DESeq2 [differential expression analysis |
|  | Metagen [meta-analysis]  GOSeq [gene ontology] | |

**Supplementary Figure 5: AS3MT gene expression-** Normalised counts for AS3MT in both RNAseq experiments shows a marked reduction in expression in the knockout (KO) lines compared to the wildtype (WT) lines. DESeq2 log2 fold change -0.566, p-adj 2.13x10^-5^, and -0.891, p-adj 2.24x10^-24^.

**
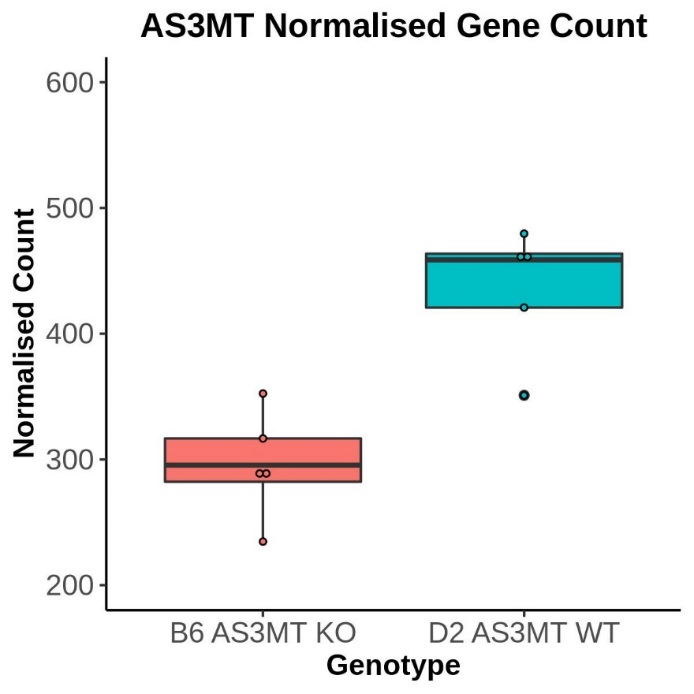

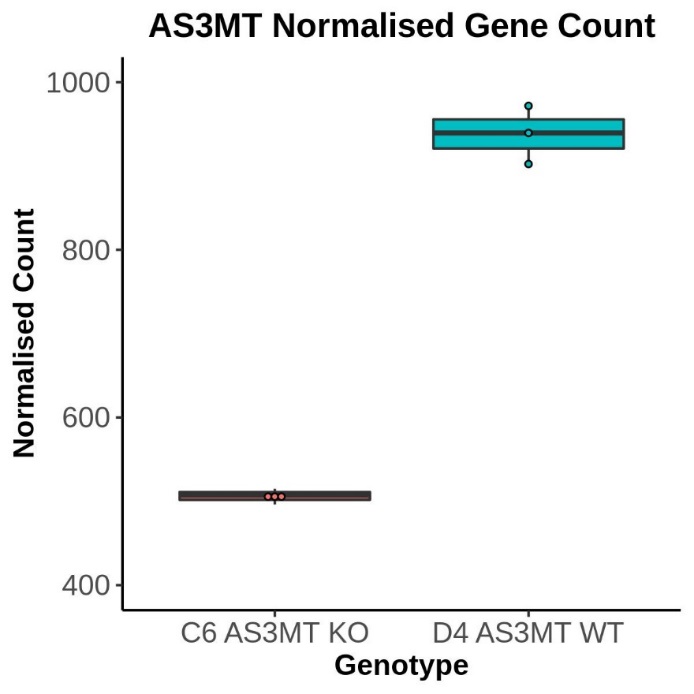
**

**Supplementary Figure 6: Differential exon usage analysis:** Expression of *BORCS7, BORCS7-AMT,* and *AS3MT* exons in both RNAseq experiments ((A) B6 KO vs D2 WT, (B) C6 KO vs D4 WT)). There is a significant reduction in expression of exon 5 and 6 of *AS3MT* (here labelled E018/E019) in the KO lines (red) compared to the WT lines (blue). B6 DEXSeq log2 fold change (Exon 5: -16.75, p-adj 9.18x10^-84^. Exon 6: -2.13, p-adj 5.89x10^-11^). C6 DEXSeq log2 fold change (Exon 5: -18.12, p-adj 4.03x10^-161^. Exon 6: -2.90, p-adj 6.31x10^-58^).


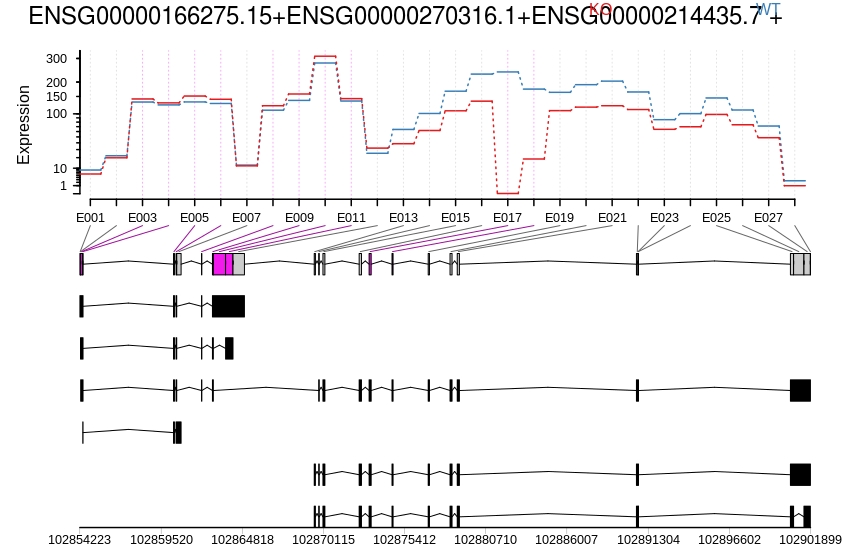

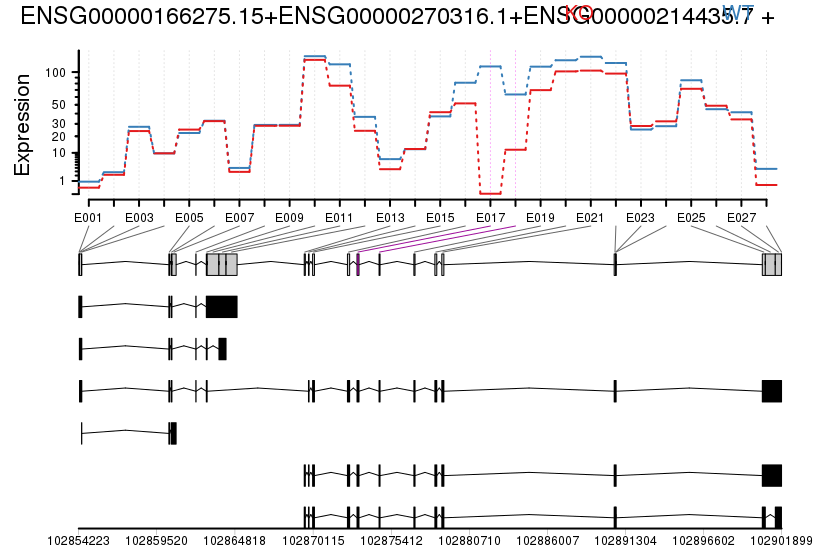


**Transcript**

ENST00000369880.8

ENST00000615257.1

*BORCS7-AMT*

*AS3MT*

*AS3MT*

*BORCS7*

*BORCS7*

*BORCS7*

**Gene**

ENST00000299353.6

ENST00000478833.1

ENST00000339834.10

ENST00000369883.3

**Transcript**

ENST00000369880.8

ENST00000615257.1

*BORCS7-AMT*

*AS3MT*

*AS3MT*

*BORCS7*

*BORCS7*

*BORCS7*

**Gene**

ENST00000299353.6

ENST00000478833.1

ENST00000339834.10

ENST00000369883.3

B

**Supplementary Figure 7**: Effect sizes of differentially expressed genes in the AS3MT B6 knockout line are correlated with those in the AS3MT C6 knockout cell line. There is a positive correlation for effect size (Log2 Fold Changes are compared to AS3MT WT cell lines). Exact binomial test, p-value=2.2x10^-16^.

A

**
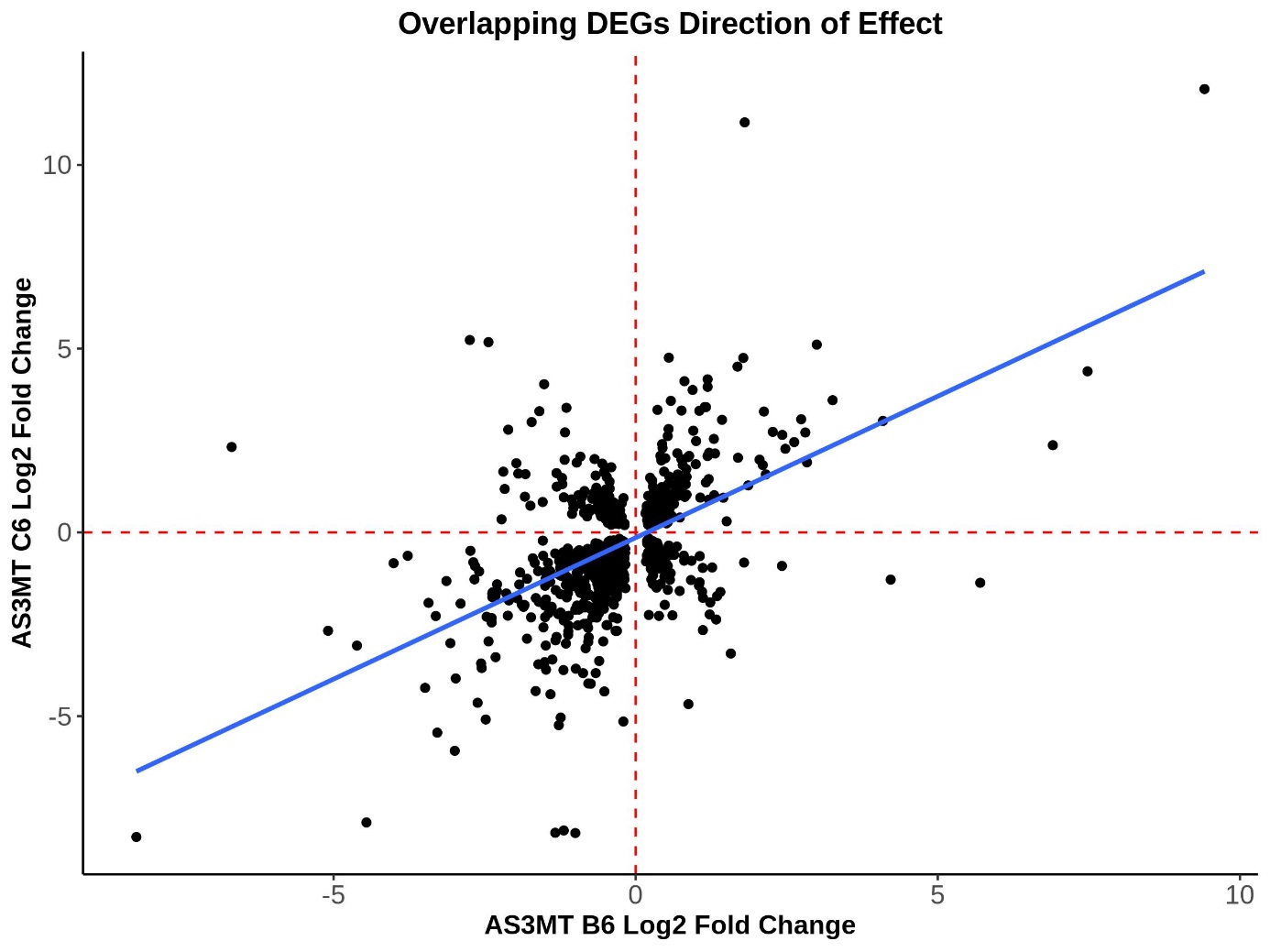
**
